# Antiproliferative and Trypanocidal Activity of Ivermectin Bioconjugates

**DOI:** 10.1021/acsomega.5c02998

**Published:** 2025-06-18

**Authors:** Michał Sulik, Dagmara Otto-Ślusarczyk, Dietmar Steverding, Marta Struga, Adam Huczyński

**Affiliations:** † Department of Medical Chemistry, Faculty of Chemistry, 49562 Adam Mickiewicz University, Uniwersytetu Poznańskiego 8, 61−614 Poznań, Poland; ‡ Chair and Department of Biochemistry, Faculty of Medicine, Medical University of Warsaw, Banacha 1, 02−097 Warsaw, Poland; § Bob Champion Research & Education Building, Norwich Medical School, University of East Anglia, Norwich NR4 7TJ, U.K.

## Abstract

Ivermectin (**IVR**), whose discovery has been Nobel-Prize-honored, is a 16-membered macrocyclic lactone used in medicine as an extremely effective and safe antiparasitic drug. In recent years, interest in this compound has grown due to its potential effectiveness in killing various types of cancer cells. However, research on the anticancer activity of **IVR** derivatives is limited. Additionally, the growing problem of drug resistance raises concerns about the effectiveness of this drug in the treatment of parasitic diseases. Therefore, in this work, we provide a detailed description of the synthesis of ten new **IVR** bioconjugates with compounds exhibiting high anticancer and/or antimicrobial activity. We also assess the effectiveness of these hybrids in killing *Trypanosoma brucei brucei* a protozoan parasite that causes African trypanosomiasis, as well as their anticancer activity toward various cancer cell lines. Many of the newly synthesized conjugates exhibited higher biological activity than their respective parent compounds as well as increased selectivity indices. The **IVR** conjugate with artesunate (compound **16**) appears particularly interesting, as it proved not only to be several times more active than the parent compounds but also showed no toxicity toward a reference cell line, indicating its potential as a therapeutic agent.

## Graphical abstract

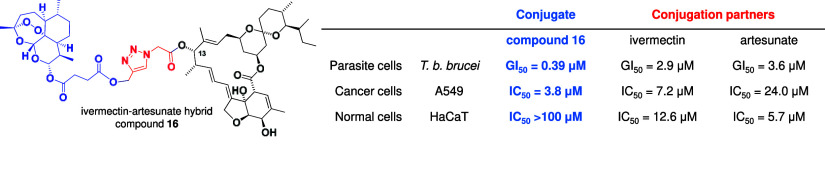

## 1 Introduction

Cancer is a global health issue that continues to be one of the leading causes of death in modern civilization. According to the World Health Organization (WHO), in 2020, approximately 10 million people died from cancer, while more than 19 million new cancer cases were diagnosed worldwide.^1,2^ Despite significant progress in modern therapeutic methods and numerous clinical studies currently conducted across the world, the search for new candidates for effective and safe anticancer drugs remains one of the most important challenges of modern science and medicine.^1,2^ Among the anticancer drugs currently in use, many classes of chemical compounds can be distinguished, each characterized by a different mechanism of biological activity. These include DNA-alkylating agents, mitosis inhibitors, and antimetabolites.^3^ Many of them–in addition to their high anticancer activity–are also highly toxic, which is often associated with the occurrence of serious side effects.^3^ One method of mitigating the unfavorable toxicity of anticancer drugs is their bioconjugation with other highly selective chemical compounds.^4,5^ Moreover, if these compounds also exhibit anticancer activity, the resulting hybrids may prove to be extremely effective in the fight against cancer, as multiple cytotoxic mechanisms will act on the cell simultaneously.

Ivermectin (**IVR**, 1, Figure 1) is a 16-membered lactone isolated in 1967 from a *Streptomyces avermitilis* strain.^6^ This compound exhibits a broad spectrum of antiparasitic activity and is used in the treatment of various parasitoses, such as onchocerciasis, lymphatic filariasis, and scabies.^7,8^ It is used as a mixture of two homologues: B1a, which has an ethyl group at position C26, and B1b, which has a methyl group at this position.^6,9^ The mixture consists of at least 80% B1a and no more than 20% of B1b.^6,9^ Due to its potent antiparasitic activity and safety profile, **IVR** is often referred to as a “wonder drug”.^6,8,10^ Its discovery was honored with the Nobel Prize in Physiology or Medicine in 2015.^11^ Recent studies have shown that, besides its great potential in combating different parasites, this drug exhibits high anticancer activity against various types of cancer cell lines, including glioma, leukemia, pancreatic cancer, and colon cancer.^12^ The anticancer mechanism of action of **IVR** is much more complex and involves many different biochemical processes, including the inhibition of the synthesis of multidrug resistance (MDR) proteins or the increase in reactive oxygen species (ROS) levels.^12,13^ However, the data regarding the anticancer activity of **IVR** derivatives remain limited to only one publication, demonstrating that chemical modification at position C13 can lead to analogs with increased antiproliferative activity and/or improved selectivity.^14^ In addition to these findings, **IVR** is a safe compound with virtually no side effects and, therefore, is an extremely interesting candidate for bioconjugation.

**Figure 1:**
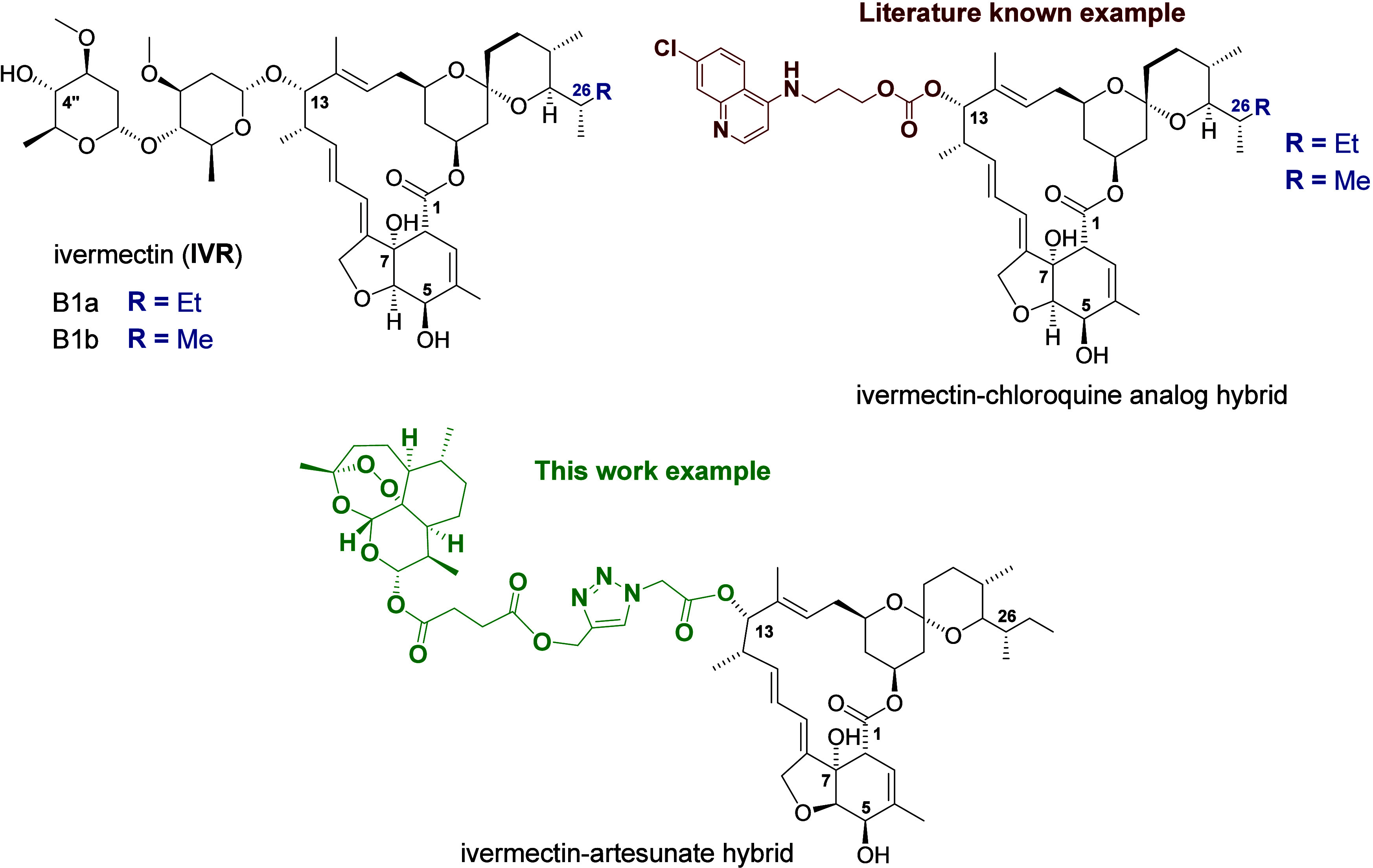
Chemical structure of ivermectin (**IVR**), its C13-hybrid with a chloroquine analog, and the C13-hybrid of **IVR** and artesunate studied in this work.

The process of bioconjugation involves the linking of two or more bioactive molecules through covalent bonding.^4,5^ It is one of the most often used strategies in developing new hybrids with higher efficacy compared with that of the parent compounds. Bioconjugates can be created by combining two or more biomolecules in a single molecule or by linking biomolecules with synthetic small molecules.^4,5^ This technique was used for the chemical modification of **IVR** by synthesizing dual-acting hybrids with different conjugation partners, such as aminoquinolines (Figure 1) or ferrocene derivatives.^15,16^ These compounds showed high in vitro activity against the blood-stage and liver-stage of *Plasmodium* parasites, confirming their potential in combating malaria.^15,16^ Linkers with diverse structures and stabilities, such as carbonates, urethanes, or the 1,2,3-triazole ring formed through copper­(I)-catalyzed alkyne–azide cycloaddition (CuAAC) between two conjugation partners, were employed in the synthesis of the respective hybrids.^15,16^

Inspired by the results regarding the anticancer properties of C13 derivatives of **IVR** and the antimalarial properties of hybrids of this drug, we decided to combine both research directions. Herein, we present synthetic access to a series of ten **IVR** bioconjugates with other biologically active components, combined using appropriate linkers (Figure 1). Compounds exhibiting diverse biological activities were selected as conjugation partners. These included cinchona bark alkaloids (**quinine**, **quinidine**, **cinchonine**, and **cinchonidine**), which have been used for many years as antimalarial and antiarrhythmic drugs, but their effectiveness in overcoming drug resistance in cancer cells has also been documented.^17,18^ In addition to these compounds, we also chose two nucleoside analogs (**floxuridine** and **azidothymidine**), which are used as anticancer drugs but are characterized by high toxicity.^19,20^ Furthermore, we also included in the study *N* **-deacetylthiocolchicine**, **betulinic acid**, **artesunate**, and **metronidazole**, known for their potent biological activities (antimicrobial and/or anticancer).^21–25^

All newly synthesized conjugates were evaluated in vitro for their cytotoxic activity toward different cell lines. However, it should be mentioned that the primary use of **IVR** is as an extremely effective drug to treat various parasitic diseases.^8^ Moreover, many of the conjugation partners named above are also highly effective against parasites. Thus, the resulting hybrids may exhibit potent antiparasitic activities, as well. To test the antiparasitic properties of the bioconjugates, we selected *Trypanosoma brucei*, the causative agent of African trypanosomiasis, a parasite belonging to the group of organisms causing neglected tropical diseases.^26–28^ It should also be noted that C13 derivatives of **IVR** display promising activity against *T. brucei*.^29^ Thanks to preventive and new therapeutic interventions introduced by the World Health Organization (WHO), the number of human African trypanosomiasis (sleeping sickness) cases has been significantly reduced in recent years, while animal African trypanosomiasis (nagana disease) remains a problem in sub-Saharan Africa.^28,30^ However, due to the limited number of drugs and the emergence of drug-resistant parasites, the search for new bioactive compounds to combat this disease is necessary.^31^ Therefore, all newly synthesized hybrids were also tested for their in vitro activity against bloodstream forms of *T. b. brucei*.

## 2 Results and Discussion

### 2.1 Analogs Design and Synthesis

A characteristic feature of **IVR** is the presence of a disaccharide moiety at the C13 position. It has been shown that its presence is not necessary for the antiparasitic activity of the drug, but the aglycone exhibits generally poorer biological properties than the unmodified **IVR**.^32,33^ An interesting direction for research is, therefore, the replacement of the sugar moiety with other chemical groups. Thus, the synthesis of C13-hybrids of **IVR** with compounds showing anticancer and/or antimicrobial activity was conducted. Linkers with diverse chemical properties were used, including carbonates, urethane, and 1,2,3-triazole rings. The reactions involved conjugation partners in their unmodified form or after structural modifications. In general, three methods of bioconjugate synthesis were employed.

The first method involved the use of triphosgene, enabling the conjugation of **IVR** with cinchona bark alkaloids and *N*-deacetylthiocolchicine. The cinchona bark alkaloids were used in their native form, while *N*-deacetylthiocolchicine had to be synthesized. It has been shown that the substitution of colchicine’s C10-methoxy group with a thiomethyl group increases the molecular stability of the resulting thiocolchicine.^21^ As further reaction required the presence of a free amine group, a deacetylation of thiocolchicine was carried out, resulting in *N*-deacetylthiocolchicine, which was then used for subsequent bioconjugation (Scheme 1).^21^

**Scheme 1:**
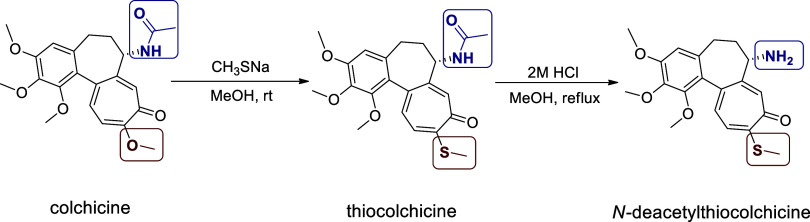
Synthesis of *N*-Deacetylthiocolchicine

The bioconjugation reaction was carried out between the appropriate partners and the C5-protected aglycone of **IVR** (compound **3**, Scheme 2). Compound **3** was obtained through a two-step reaction, which included the solvolysis of the sugar moiety in the presence of sulfuric acid and the protection of the C5-hydroxyl group using *tert*-butyldimethylsilyl chloride (Scheme 2). The reaction between the C13-hydroxyl group of compound **3** and the hydroxyl group of cinchona bark alkaloids, or the amine group of *N*-deacetylthiocolchicine, took place in the presence of triphosgene, which enabled the synthesis of either a carbonate or urethane linker. Deprotection of position C5 using *p*-toluenesulfonic acid allowed the formation of hybrids **4–8** with overall yields from two steps (bioconjugation and deprotection) ranging from 14 to 21% (Scheme 2).

**Scheme 2:**
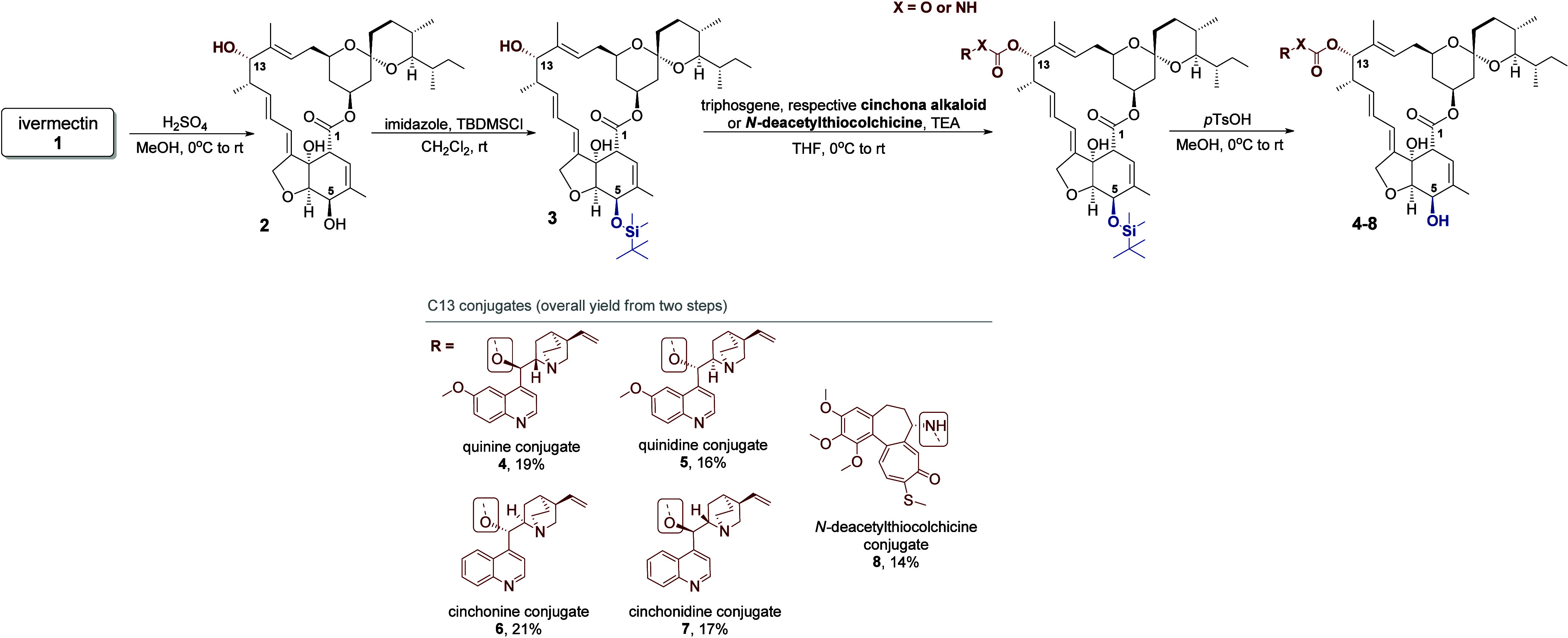
Synthesis of Bioconjugates of **IVR** with Cinchona Bark Alkaloids and *N*-Deacetylthiocolchicine

The second method allowed the conjugation of the **IVR** skeleton with nucleoside analogs (floxuridine and azidothymidine) and metronidazole. Similarly to the first method, the linker was a carbonate group; however, the use of triphosgene proved unsuitable for these compounds, as no formation of the expected product was observed. Therefore, we decided to use carbonyldiimidazole (CDI) as a conjugative agent, which was proposed by Singh et al. in 2022 for the synthesis of **IVR** conjugates with aminoquinolines (Scheme 3).^16^ For this purpose, C5-protected compound **3** was subjected to a reaction with CDI, which allowed the synthesis of precursor **9**. This reagent was then added to a mixture of nucleoside analogue or metronidazole with DBU. The reaction was conducted at 90 °C, and the isolation and subsequent deprotection of the C5-hydroxyl group resulted in the desired hybrids **10–12**, with yields ranging from 15 to 30% (Scheme 3).

**Scheme 3:**
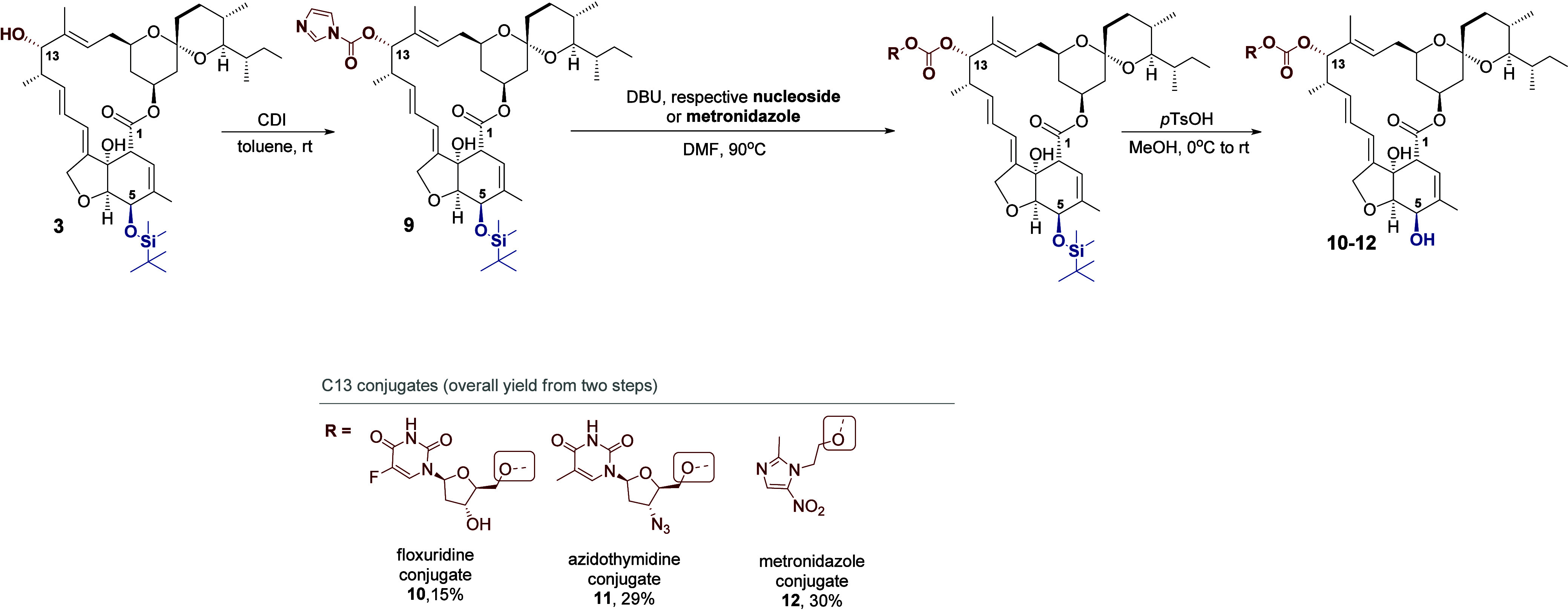
Synthesis of Bioconjugates of **IVR** with Nucleoside Analogs (Floxuridine and Azidothymidine) and Metronidazole

The third method allowed the conjugation of **IVR** with betulinic acid and artesunate through the use of the CuAAC reaction under Meldal conditions (copper­(I) iodide as the source of Cu­(I) ions).^34^ To facilitate this, it was necessary to prepare the azide and propargyl partners to enable the click reaction. Propargyl ester of betulinic acid was obtained by reacting betulinic acid with propargyl bromide in the presence of DBU. However, these conditions were unsuitable for the synthesis of the propargyl ester of artesunate, as they led to the decomposition of the starting material. Consequently, to obtain the ester, propargyl alcohol was used in the presence of DCC and DMAP (Scheme 4).

**Scheme 4:**
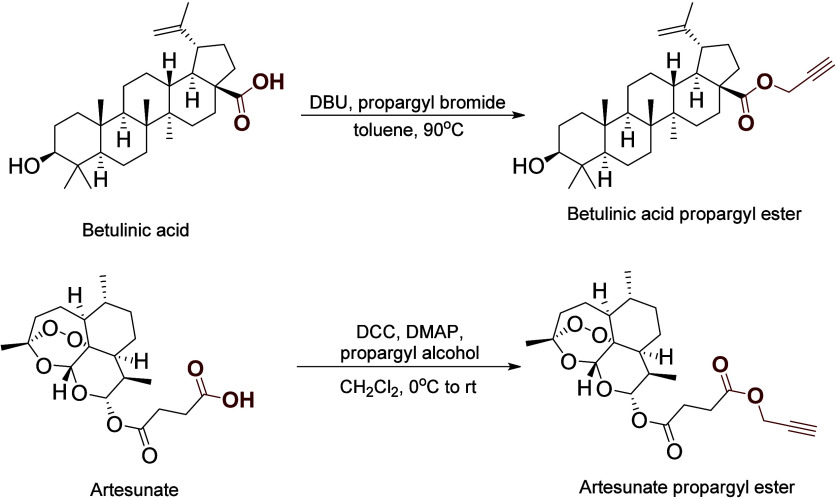
Synthesis of Propargyl Esters of Betulinic Acid and Artesunate

To prepare the C13-azide precursor of **IVR**, the procedure described by Singh et al. was employed.^15^ It involved the synthesis of a chloroacetyl ester **13** formed by the reaction between compound **3** and chloroacetyl chloride (Scheme 5). This ester was then subjected to a nucleophilic substitution reaction with sodium azide (Scheme 5). The reaction did not proceed quantitatively, and the resulting azide **14** was difficult to separate chromatographically. Therefore, the click reaction was carried out using a mixture of azide **14** and substrate **13**.

**Scheme 5:**
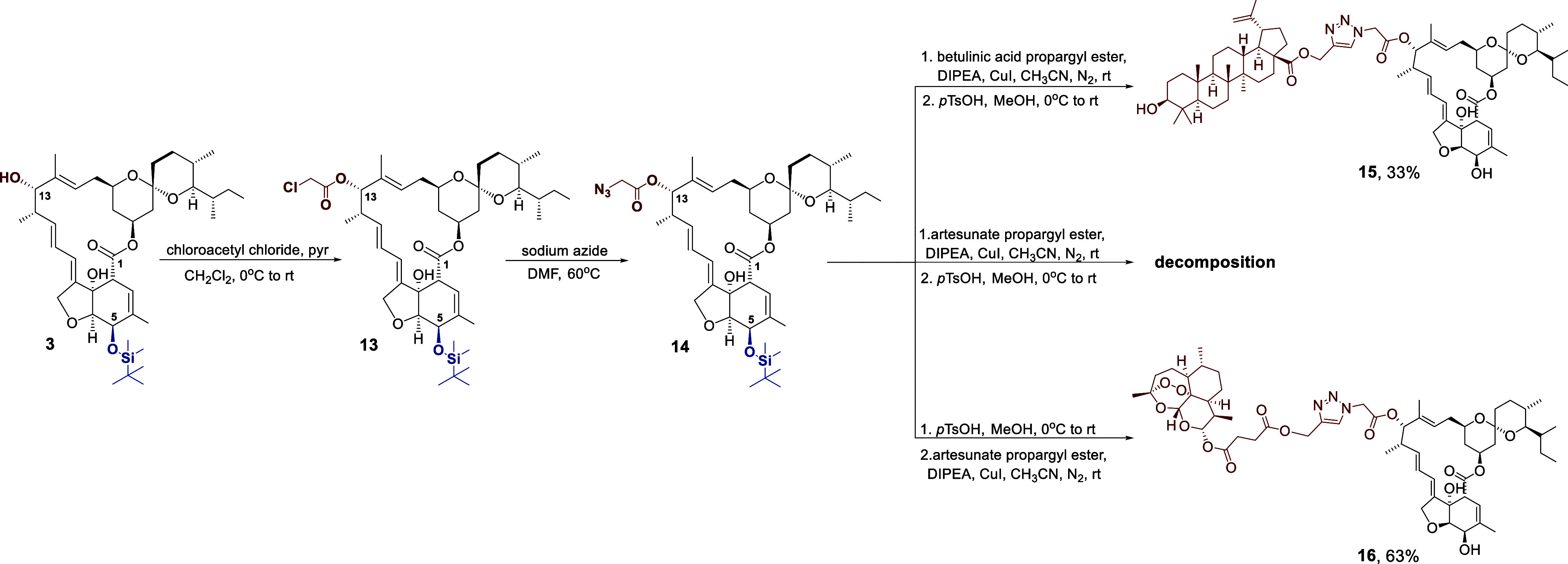
Synthesis of Bioconjugates of **IVR** with Betulinic Acid and Artesunate

The obtained precursors (azide **14** and the respective ester) were dissolved in acetonitrile, and then DIPEA and copper­(I) iodide were added. The reactions were carried out in an inert gas atmosphere to avoid oxidation of copper­(I) iodide. Deprotection of position C5 was then carried out using *p*-toluenesulfonic acid (Scheme 5). This method proved suitable for the synthesis of the **IVR**/betulinic acid bioconjugate (compound **15**, yield 33%), but it led to the decomposition of artesunate. Therefore, to obtain compound **16**, it was necessary to reverse the synthetic procedure (Scheme 5). Azide **14** was first subjected to deprotection with *p*-toluenesulfonic acid and then used in a click reaction with the propargyl ester of artesunate. This approach allowed the synthesis of compound **16** with a click reaction yield of 63% (Scheme 5).

Purity and structure of the synthesized **IVR** bioconjugates were determined using spectroscopic (^1^H NMR, ^13^C NMR) and spectrometric (ESI-MS) methods. In the ^13^C NMR spectra of **IVR** bioconjugates, the signals of the highest analytical significance were assigned to the newly introduced carbonyl group of the carbonate, urethane, or ester moiety at position C13. Depending on the type of substituent, the signal from the ester group appeared in the range of 176.3–165.3 ppm. The signals from the carbonate group were detected in the range of 153.9–154.6 ppm, while the signal from the urethane group of compound **8** appeared at 158.4 ppm. The signals from the lactone group at position C1 were observed in the range of 173.4–168.3 ppm. In the ^1^H NMR spectra of the click derivatives (compounds **15** and **16**), as well as metronidazole bioconjugate **12**, a characteristic, intensive singlet originating from the hydrogen atom of the triazole/imidazole ring emerged in the narrow range of 7.88–7.78 ppm. The ESI-MS analysis confirmed the formation of the desired products, with [M + Na]^+^ or [M + H]^+^ as the main peak (intensity = 100%). The NMR and ESI-MS spectra of all novel **IVR** derivatives are included in the Supporting Information (Figures S1–S23).

### 2.2 Biological Activity

#### 2.2.1 Trypanocidal Activity

The trypanocidal and cytotoxic activities of the newly synthesized **IVR** bioconjugates and their conjugation partners were determined for bloodstream forms of *T. brucei brucei* and human myeloid HL-60 cells in vitro using the resazurin cell viability assay as previously described.^29^ For most bioconjugates (**4–12**), the trypanocidal activity was found to be only slightly better than that of **IVR**, with 50% growth inhibition (GI_50_) values ranging between 1.3–2.4 μM (Table 1). In the case of bioconjugates **4**, **5**, **6**, **7**, and **10**, the observed antitrypanosomal activity was between that of **IVR** and the corresponding conjugation partner. As *N* **-deacetylthiocolchicine**, **azidothymidine**, and **metronidazole** displayed trypanocidal activities with GI_50_ values >10 μM (Table 1), it may be suggested that these conjugation partners can enhance the trypanocidal activity of **IVR**. The antitrypanosomal activity of the bioconjugate **15** (GI_50_ = 21.9 μM) was lower than that of **IVR** and its conjugation partner (Table 1), suggesting that the combination of **IVR** and betulinic acid reduces their individual effectiveness when linked together. An encouraging result was obtained for bioconjugate **16** (GI_50_ = 0.39 μM) as its antitrypanosomal activity was 7 times and 9 times stronger than those of **IVR** and **artesunate**, respectively (Table 1), indicating synergistic action of the two partners. Since the trypanocidal activity of an equimolar mixture of **IVR** and **artesunate** was 23 times lower than that of **16** (Table 1), it can be concluded that **IVR** and artesunate act synergistically only if linked together. The cytotoxic activity of the bioconjugates was found to be lower compared with their trypanocidal activity (Table 1). Bioconjugates **6** and **15** displayed the strongest and lowest cytotoxic activity with GI_50_ values of 4.0 and >100 μM, respectively (Table 1). Except for **7** and **15**, all other bioconjugates exhibited lower GI50 values towards HL-60 cell line than **IVR** (Table 1). All conjugation partners displayed low (**betulinic acid** and **artesunate**) or no cytotoxic activity (Table 1).

**Table 1:** GI_50_ Values and Ratios of **IVR** Conjugates and Conjugation Partners for *T. brucei* and HL-60 Cells

	*T. brucei*	HL-60	selectivity		*T. brucei*	HL-60	selectivity
compound	GI_50_ (μM)^a^	GI_50_ (μM)^a^	GI_50_ ratio^b^	compound	GI_50_ (μM)^a^	GI_50_ (μM)^a^	GI_50_ ratio^b^
**1 (IVR)**	2.9 ± 0.1	30.0 ± 1.1	10.3				

bioconjugates				conjugation partner			
**4**	2.1 ± 0.3	21.7 ± 11.4	10.3	**quinine**	1.1 ± 0.3	>100	>90.9
**5**	1.3 ± 0.4	22.9 ± 11.2	17.6	**quinidine**	0.32 ± 0.04	>100	>312.5
**6**	1.8 ± 0.4	4.0 ± 1.0	2.2	**cinchonine**	1.7 ± 0.6	>100	>58.8
**7**	2.3 ± 0.3	47.2 ± 3.7	20.5	**cinchonidine**	2.1 ± 0.6	>100	>47.6
**8**	1.4 ± 0.5	10.1 ± 4.9	7.2	***N*** **-deacetylthiocolchicine**	17.4 ± 4.3	>100	>5.7
**10**	2.4 ± 0.5	19.1 ± 9.1	8.0	**floxuridine**	2.2 ± 0.4	>100	>45.5
**11**	2.0 ± 0.8	11.1 ± 4.1	5.6	**azidothymidine**	19.1 ± 8.1	>100	>5.2
**12**	2.3 ± 0.3	9.2 ± 3.4	4.0	**metronidazole**	>100	>100	∼1.0
**15**	21.9 ± 2.1	>100	>4.6	**betulinic acid**	15.4 ± 4.4	42.5 ± 2.5	2.8
**16**	0.39 ± 0.2	18.0 ± 6.0	46.2	**artesunate**	3.6 ± 0.2	30.8 ± 9.0	8.6
IVR + artesunate	9.0 ± 3.5						
**suramin** ^c^	0.039 ± 0.004	>100	>2564				
**ethidium bromide** ^c^	0.046 ± 0.008	9.3 ± 2.6	202				

a Data shown are mean values ± SD of three independent experiments.

b GI_50_ ratio = GI_50_(HL-60)/GI_50_ (*T. brucei*).

c Reference controls.

The selectivity index (ratio of cytotoxic to trypanocidal activity) of all bioconjugates was found to be in the range of moderate values (<100) (Table 1). Only compounds **5**, **7**, and **16** had a better selectivity index than **IVR**, with **16** having the best one due to its increased trypanocidal activity (Table 1). On the other hand, the cinchona bark alkaloids (**quinine**, **quinidine**, **cinchonine**, and **cinchonidine**) had a better selectivity index than bioconjugate **16**, which can be attributed to their noncytotoxicity (GI_50_ > 100 μM, Table 1). Encouraging, however, is the finding that **16** is nontoxic against normal cells (HaCaT, Table 2). Using the cytotoxicity of normal cells (HaCaT) as the reference value, the selectivity index for 16 would then be >256. As the commercial drugs suramin and ethidium bromide (homidium bromide) used for the treatment of human and animal African trypanosomiasis, respectively, display 10 times higher trypanocidal activity, their selectivity indices are much higher (>200) (Table 1).

**Table 2:** Antiproliferative Activity (IC_50_, μM) of **IVR**, its Conjugates, and Conjugation Partners^a^

	cancer cells								normal cells
	PC3		MDA-MB-231		A549		HCT-116		HaCaT
compound	IC_50_	SI^b^	IC_50_	SI^b^	IC_50_	SI^b^	IC_50_	SI^b^	IC_50_
**1** (**IVR**)	33.9 ± 6.3	0.4	8.7 ± 3.2	1.4	7.2 ± 1.4	1.8	9.9 ± 1.1	1.3	12.6 ± 4.3
**4**	13.1 ± 3.5	2.0	16.3 ± 4.9	1.6	56.3 ± 5.1	0.5	16.7 ± 2.3	1.6	26.8 ± 4.9
**5**	12.4 ± 2.1	1.2	10.1 ± 1.4	1.5	42.3 ± 0.5	0.4	13.8 ± 3.5	1.1	14.9 ± 1.6
**6**	4.1 ± 1.7	3.6	13.8 ± 1.6	1.1	35.3 ± 6.5	0.4	12.2 ± 2.8	1.2	14.6 ± 1.6
**7**	7.2 ± 3.8	7.0	22.4 ± 1.2	2.2	13.6 ± 1.1	3.7	20.1 ± 3.7	2.5	50.3 ± 4.9
**8**	85.1 ± 9.8	>1.2	6.6 ± 0.6	>15.2	12.4 ± 2.6	>8.1	7.5 ± 1.6	>13.3	>100
**10**	7.8 ± 2.9	2.0	13.2 ± 1.1	1.2	15.5 ± 3.2	1.0	14.3 ± 1.9	1.1	15.4 ± 1.1
**11**	8.1 ± 3.2	3.2	12.5 ± 1.5	2.1	8.2 ± 1.9	3.2	7.7 ± 3.1	3.4	26.1 ± 3.3
**12**	5.9 ± 1.7	4.5	13.6 ± 3.7	2.0	13.2 ± 3.4	2.0	9.1 ± 0.6	2.9	26.7 ± 2.6
**15**	93.9 ± 6.4	>1.1	>100	∼1.0	>100	∼1.0	70.1 ± 6.4	>1.4	>100
**16**	20.8 ± 7.2	>4.8	13.0 ± 4.1	>7.7	3.8 ± 1.3	>26.3	4.5 ± 0.6	>22.2	>100
**quinine**	56.3 ± 7.2	>1.8	54.4 ± 7.0	>1.8	56.4 ± 3.2	>1.8	32.8 ± 3.8	>3.0	>100
**quinidine**	86.1 ± 1.2	>1.2	76.2 ± 4.6	>1.3	55.8 ± 5.3	>1.8	67.4 ± 8.9	>1.5	>100
**cinchonine**	74.3 ± 5.4	>1.3	65.3 ± 2.8	>1.5	>100	∼1.0	78.2 ± 5.8	>1.3	>100
**cinchonidine**	86.7 ± 3.9	>1.2	>100	∼1.0	>100	∼1.0	87.1 ± 12.1	>1.1	>100
*N* **-deacetylthiocolchicine**	56.1 ± 2.5	1.6	84.2 ± 7.1	1.0	>100	<0.9	70.3 ± 5.9	1.2	87.4 ± 10.4
**floxuridine**	15.7 ± 3.3	1.5	22.2 ± 3.4	1.1	26.4 ± 3.8	0.9	11.7 ± 4.2	2.1	24.3 ± 7.3
**azidothymidine**	64.3 ± 3.7	>1.6	52.2 ± 4.1	>1.9	72.1 ± 11.6	>1.4	60.2 ± 9.7	>1.7	>100
**metronidazole**	57.0 ± 6.1	>1.8	14.9 ± 3.9	>6.7	>100	∼1.0	>100	∼1.0	>100
**betulinic acid**	66.6 ± 2.3	>1.5	46.5 ± 4.6	>2.2	98.1 ± 3.1	>1.0	80.1 ± 6.1	>1.2	>100
**artesunate**	48.5 ± 5.7	0.1	12.6 ± 3.9	0.5	24.0 ± 6.9	0.2	37.8 ± 4.2	0.2	5.7 ± 0.1
**IVR + artesunate**	1.8 ± 0.5	8.9	22.5 ± 2.6	0.7	2.4 ± 0.7	6.7	1.7 ± 0.2	9.5	16.1 ± 2.9
**doxorubicin** ^c^	0.6 ± 0.02	0.5	1.83 ± 0.1	0.2	0.63 ± 0.2	0.5	0.6 ± 0.02	0.5	0.29 ± 0.1

a Data are expressed as mean ± SD; IC_50_ (μM), the concentration of the compound that corresponds to a 50% growth inhibition of the cell line (compared to the control) after culturing the cells for 72 h with the individual compound; PC3, human metastatic prostate cancer cell line; MDA-MB-231, human triple-negative breast cancer cell line; A549, human lung cancer cell line; HCT-116, human primary colon cancer cell line; HaCaT, human immortalized keratinocyte cell line.

b SI (selectivity index) was calculated using the formula: SI = IC_50_ for normal cell line (HaCaT)/IC_50_ for respective cancer cell line (PC3, MDA-MB-231, A549, or HCT-116). Briefly, an SI > 1.0 indicates that the compounds exhibit greater potency against cancer cells than toxicity toward nontumor cells.^35^

c Reference compound commonly used in cancer treatment.

#### 2.2.2 Antiproliferative Activity

The antiproliferative activities of **IVR**, its newly synthesized bioconjugates, and its precursors were evaluated against four human cancer cell lines: PC3 (metastatic prostate cancer), MDA-MB-231 (triple-negative breast adenocarcinoma), A549 (lung cancer), and HCT-116 (primary colon cancer) using the MTT colorimetric assay (Table 2). Additionally, to assess the selectivity of these compounds toward cancer cells, we included an immortalized keratinocyte cell line from adult human skin (HaCaT) in the antiproliferative activity tests. Doxorubicin served as the reference anticancer drug. First, it is important to highlight the antiproliferative activity exhibited by **IVR**. This drug demonstrates the highest activity among all compounds used for bioconjugation across all tested cell lines, with the exception of **floxuridine**, which displays a lower IC_50_ value than **IVR** for the PC3 cell line (Table 2). However, it should be noted that **IVR** exhibits the highest cytotoxicity toward the HaCaT cell line among all compounds used for bioconjugation, except **artesunate**. Despite that, **IVR** selectively targets cancer cells from the MDA-MB-231, A549, and HCT-116 lines more effectively than the reference HaCaT cell line (Table 2). Consequently, the selectivity indices of **IVR** are comparable to those of the other conjugation partners. Second, the bioconjugates had, in most cases, lower biological activity than **IVR**. The **IVR** bioconjugates with cinchona bark alkaloids (compounds **4–8**) generally showed reduced antiproliferative activity compared to unmodified **IVR** (Table 2). However, an interesting result was observed for the PC3 cell line, which was particularly resistant to the effects of **IVR** and cinchona bark alkaloids. The obtained bioconjugates are effective against the PC3 cancer cell line, with **6** showing an IC_50_ value of 4.1 μM, making it one of the most active compounds in the entire series of bioconjugates (Table 2).

Furthermore, most **IVR** bioconjugates with cinchona bark alkaloids showed satisfactory selectivity index values, with compound **7** reaching a value of 7.0 (Table 2). Although *N* **-deacetylthiocolchicine** exhibited low bioactivity across all tested cancer cell lines, the corresponding bioconjugate (compound **8**) had IC_50_ values comparable to those of unmodified **IVR**. In addition, compound **8** showed high selectivity, as it was completely nontoxic to the reference HaCaT cell line (SI > 15.2 for the MDA-MB-231 cell line). **IVR** bioconjugates with the nucleoside analogs and **metronidazole** (compounds **10–12**) exhibited the highest antiproliferative activity against the PC3 cancer cell line (IC_50_ = 5.9–8.1 μM), comparable to that of cinchona bark alkaloids, which indicates that the replacement of the sugar moiety at position C13 of **IVR** with small-molecular conjugation partners enhanced their activity against the PC3 cell line (Table 2).

Bioconjugates with partners characterized by high molecular weight and greater steric hindrance, such as *N*-**deacetylthiocolchicine** or **betulinic acid**, seemed to be ineffective against cancer cells of the PC3 line. Moreover, the **IVR** bioconjugate with **betulinic acid** (compound **15**) proved to be the least active compound from the entire series (Table 2). This is likely due to its low bioavailability, as this hybrid showed no activity against either cancer cell lines or the reference cell line. A particularly promising compound was the **IVR** bioconjugate with **artesunate** (compound **16**). This hybrid proved to be not only the most active bioconjugate from the entire series (IC_50_ = 3.8 μM against the A549 cell line and IC_50_ = 4.5 μM against the HCT-116 cell line), but it also showed no toxicity against the HaCaT cell line (Table 2). This indicates that compound **16** is highly selective (SI > 26.3 against the A549 cell line) and could be a lead compound for further drug development.

Inspired by the high potential of compound **16**, we evaluated the antiproliferative activity of an equimolar mixture of **IVR** and **artesunate** (Table 2). As shown, the results are ambiguous. In the case of the PC3 cell line, the mixture of the two compounds exhibited significantly greater activity compared to that of compound **16, IVR**, and **artesunate** tested individually (Table 2). For the A549 and HCT-116 cell lines, the mixture and the bioconjugate **16** demonstrated comparable levels of activity, slightly in favor of the mixture. In contrast, for the MDA-MB-231 cell line, compound **16** showed superior antiproliferative activity relative to that of the drug combination. Despite these inconsistencies, it is important to note the differences in cytotoxicity with respect to the HaCaT reference cell line. Bioconjugation of the two agents effectively mitigated the negative effects of both drugs on noncancerous cells, underscoring the beneficial outcomes of the bioconjugation strategy.

Finally, although doxorubicin showed strong antiproliferative activity against all tested cancer cell lines, it is important to emphasize its high cytotoxicity toward HaCaT cells (Table 2). In all cases, the SI values of doxorubicin were lower than 1.0, indicating that the compound preferentially targets healthy cells over cancerous ones (Table 2). In contrast, almost all bioconjugates displayed SI values greater than 1.0, underscoring their high anticancer potential (Table 2).

## 3 Conclusions

A series of new **IVR** bioconjugates with partners exhibiting high antitumor or antimicrobial activity have been synthesized. These partners included four cinchona bark alkaloids, nucleoside analogs, metronidazole, *N*-deacetylthiocolchicine, betulinic acid, and artesunate. The hybrids were obtained using three different synthetic procedures, allowing the introduction of various linkers such as carbonate, urethane, or a 1,2,3-triazole ring. All obtained bioconjugates, along with their respective conjugation partners, were evaluated in vitro for their activity against the protozoan parasite *T. b. brucei*. For most bioconjugates, the trypanocidal activity was slightly better than that of **IVR,** with GI_50_ values ranging between 1.3–2.4 μM. For the bioconjugates **4**, **5**, **6**, **7**, and **10**, the antitrypanosomal activity was between that of **IVR** and the corresponding conjugation partner. In addition to the antitrypanosomal studies, in vitro experiments were also conducted against various cancer cell lines. It has been shown that the conjugation of **IVR** with cinchona bark alkaloids, nucleosides, or metronidazole is an effective method to enhance the antiproliferative activity of **IVR** against the PC3 cancer cell line, which was most resistant to the action of **IVR** and the conjugation partners. On the other hand, compound **8** exhibited antiproliferative activity similar to that of **IVR**, but its lack of toxicity against the HaCaT reference cell line suggests that it is a highly selective anticancer agent. In both antiparasitic and anticancer activity assays, compound **16** (**IVR**-artesunate bioconjugate) showed the highest activity. This hybrid was not only several times more active than its components but also demonstrated low toxicity against the reference cell line. Moreover, trypanocidal studies of the **IVR**-artesunate equimolar mixture revealed that the synergistic effect of the combination of these two drugs was observed only after conjugation. A mixture of the two compounds acts antagonistically, showing lower activity than **IVR** or artesunate alone. Analogous antiproliferative studies gave more variable results; however, compound **16** proved to be significantly less cytotoxic against noncancerous cells than the combination of **IVR** and artesunate. These observations confirm that conjugation of **IVR** is a promising approach to develop new bioactive compounds with potential applications in anticancer and antiparasitic therapies.

## 4 Experimental Section

### 4.1 General Procedure

Ivermectin (**IVR**, form B1a) was purchased from Trimen Chemicals S.A. All other reagents were commercially available and purchased from two sources, Merck or Trimen Chemicals S.A., and were used without further purification. A detailed description of the general procedures, measurement parameters, and equipment can be found in the Supporting Information.

### 4.2 Synthesis of Ivermectin Aglycone (Compound **2**)

**IVR** (4.95 g, 5.66 mmol, 1.0 equiv) was dissolved in methanol (79.2 mL) and the solution was cooled in an ice bath. Concentrated sulfuric acid (0.8 mL) was then added dropwise to the reaction mixture. The solution changed color to greenish, and after a few hours, it turned yellow. After 20 h, the reaction mixture was concentrated by using a rotary evaporator, diluted with CH_2_Cl_2_, and extracted with an aqueous sodium carbonate solution (0.1 M). The organic layers were then concentrated under reduced pressure. The product was purified on silica gel using the CombiFlash system (0% → 30% EtOAc/CHCl_3_), yielding pure aglycone **2** as a clear oil. After two evaporation steps with *n*-pentane to remove any remaining solvent, the oily product was fully converted to a white, amorphous solid (2.79 g, 84% yield). The spectroscopic data were in agreement with previously published data.^15^

### 4.3 Protection of the C5 Hydroxyl Group of Ivermectin Aglycone (Compound **3**)

To a stirred solution of **2** (2.00 g, 3.41 mmol, 1.0 equiv) in anhydrous CH_2_Cl_2_ (40 mL), imidazole (2.32 g, 34.07 mmol, 10.0 equiv) was added. Once the imidazole had dissolved, *tert*-butyldimethylsilyl chloride (1.13 g, 7.50 mmol, 2.2 equiv) was added in one portion. The reaction mixture was stirred at room temperature for 24 h. After this period, the reaction mixture was concentrated to dryness using a rotary evaporator. The product was then purified on silica gel using the CombiFlash system (0% → 10% EtOAc/CHCl_3_), yielding pure product **3** as a clear oil. After two evaporation steps with *n*-pentane to remove residual solvent, the oily product was fully converted to a yellow amorphous solid (2.17 g, 91% yield). The spectroscopic data were in agreement with previously published data.^15^

### 4.4 Synthesis of *N*-Deacetylthiocolchicine

To a mixture of colchicine (500 mg, 1.25 mmol) in MeOH (5 mL) was added a sodium methanethiolate solution (21% in H_2_O, 0.83 mL, 2.5 mmol). The mixture was stirred at room temperature, and the reaction progress was monitored by TLC. After 24 h, the reaction mixture was quenched by adding water (150 mL). The mixture was then extracted four times with CH_2_Cl_2_, and the combined organic layers were dried over MgSO_4_, filtered, and evaporated under reduced pressure. The residue was purified by the CombiFlash system (EtOAc/MeOH, increasing concentration gradient) to yield thiocolchicine as an amorphous yellow solid (78% yield). Next, a solution of thiocolchicine (500 mg, 1.18 mmol) in MeOH (3 mL) was treated with a 2 M HCl solution (5 mL). The mixture was stirred at 90 °C for 72 h, and the reaction progress was monitored by TLC. Afterward, the solvent was evaporated under reduced pressure. The residue was purified by CombiFlash system (EtOAc/MeOH, increasing concentration gradient) to yield *N*-deacetylthiocolchicine (82% yield). The spectroscopic data were in agreement with previously published data.^21^

### 4.5 General Procedure for the Preparation of Ivermectin Conjugates with *N*-Deacetylthiocolchicine and Cinchona Bark Alkaloids (Compounds **4–8**)

Precursor **3** (400 mg, 0.57 mmol, 1.0 equiv) was dissolved in anhydrous THF (20 mL). The solution was then cooled in an ice bath, and triethylamine (288 mg, 2.85 mmol, 5.0 equiv). Next, triphosgene (85 mg, 0.29 mmol, 0.5 equiv) was added to the reaction mixture in one portion, and stirring was continued for 1 h. The formation of a white precipitate was observed. Then, the appropriate conjugation partner (*N*-deacetylthiocolchicine or cinchona bark alkaloid, 0.86 mmol, 1.5 equiv) was added to the reaction mixture, and stirring was continued for 24 h. Thereafter, the reaction mixture was concentrated to dryness using a rotary evaporator, diluted with CH_2_Cl_2_, and extracted with brine. The collected organic layers were subsequently concentrated *in vacuo*. Purification on silica gel using the CombiFlash system (0% → 100% EtOAc/CHCl_3_) gave the respective C5-protected bioconjugates as clear oils.

To remove the C5-protecting group, the individual hybrids were dissolved in methanol (10 mL) and *para*-toluenesulfonic acid hydrate (80 mg, 0.46 mmol, 1.3 equiv) was added. After 2 h, the organic solvent was evaporated, diluted with CH_2_Cl_2_, and extracted with an aqueous solution of sodium carbonate (0.1 M). Next, the collected organic layers were subsequently concentrated *in vacuo*. Purification on silica gel using the CombiFlash system (0% → 100% EtOAc/CHCl_3_) gave the pure products **4–8** as clear oils. After twice evaporation to dryness with *n*-pentane, the oily products were completely converted to white or yellow amorphous solids.

#### 4.5.1 Ivermectin–Quinine Conjugate **4**

86 mg, 19% yield (over two steps). Isolated as a white, amorphous solid, a single spot by TLC. UV-active and strains green with PMA; ^1^H NMR (401 MHz, CDCl_3_) δ 8.74 (d, *J* = 4.5 Hz, 1H), 8.02 (d, *J* = 9.9 Hz, 1H), 7.40 (d, *J* = 4.6 Hz, 1H), 7.38–7.34 (m, 2H), 6.30 (d, *J* = 5.2 Hz, 1H), 5.88–5.83 (m, 1H), 5.83–5.76 (m, 1H), 5.76–5.72 (m, 1H), 5.68 (dd, *J* = 14.7, 9.3 Hz, 1H), 5.41 (s, 1H), 5.37–5.27 (m, 1H), 5.13 (dd, *J* = 10.6, 4.9 Hz, 1H), 4.98 (dd, *J* = 13.7, 8.3 Hz, 2H), 4.87 (s, 1H), 4.66 (qd, *J* = 14.5, 2.1 Hz, 2H), 4.30–4.26 (m, 1H), 4.25–4.21 (m, 1H), 3.97–3.92 (m, 4H), 3.70–3.62 (m, 1H), 3.33 (q, *J* = 7.9 Hz, 1H), 3.26 (dd, *J* = 4.4, 2.2 Hz, 1H), 3.17 (t, *J* = 11.2 Hz, 2H), 3.04 (dd, *J* = 13.7, 10.2 Hz, 1H), 2.73–2.52 (m, 4H), 2.35–0.71 (m, 38H) ppm; ^13^C NMR (101 MHz, CDCl_3_) δ 173.4, 158.0, 154.2, 147.3, 144.7, 140.8, 137.8, 136.0, 134.3, 131.8, 126.7, 125.5, 121.9, 119.9, 118.2, 118.0, 117.8, 114.6, 101.1, 97.4, 83.1, 80.3, 79.2, 77.1, 68.4, 68.3, 67.6, 67.0, 59.3, 56.7, 55.6, 45.6, 42.6, 41.3, 39.7, 39.0, 36.8, 35.8, 35.5, 34.2, 34.1, 31.2, 29.2, 28.0, 27.6, 27.4, 23.8, 22.3, 19.9, 18.5, 17.4, 14.5, 14.0, 12.5, 11.8 ppm; ESI-MS *m*/*z*: [M + H]^+^ Calcd for C_55_H_73_N_2_O_11_^+^ 938; Found 937; [M + Na]^+^ Calcd for C_55_H_72_N_2_NaO_11_^+^ 960; Found 959.

#### 4.5.2 Ivermectin–Quinidine Conjugate **5**

102 mg, 16% yield (over two steps). Isolated as a white amorphous solid, a single spot by TLC. UV-active and strains green with PMA; ^1^H NMR (401 MHz, CDCl_3_) δ 8.69 (d, *J* = 4.6 Hz, 1H), 8.00 (d, *J* = 9.2 Hz, 1H), 7.38–7.30 (m, 3H), 6.48 (d, *J* = 5.7 Hz, 1H), 6.15–6.04 (m, 1H), 5.84 (dt, *J* = 10.7, 2.1 Hz, 1H), 5.81–5.72 (m, 1H), 5.66 (dd, *J* = 14.5, 9.8 Hz, 1H), 5.40 (s, 1H), 5.34–5.24 (m, 1H), 5.14 (s, 1H), 5.12–5.09 (m, 1H), 4.98 (dd, *J* = 10.1, 5.2 Hz, 1H), 4.93 (s, 1H), 4.65 (qd, J = 14.5, 2.1 Hz, 2H), 4.28 (d, *J* = 5.8 Hz, 1H), 4.25 (s, 1H), 3.95 (d, *J* = 6.1 Hz, 1H), 3.92 (s, 3H), 3.63–3.54 (m, 1H), 3.29–3.21 (m, 2H), 3.15 (d, *J* = 7.3 Hz, 1H), 2.94 (d, *J* = 9.0 Hz, 2H), 2.90–2.83 (m, 1H), 2.75 (dt, *J* = 13.3, 8.7 Hz, 1H), 2.67–2.57 (m, 2H), 2.28 (dd, *J* = 16.5, 8.5 Hz, 1H), 2.21–2.08 (m, 3H), 2.06–0.66 (m, 34H) ppm; ^13^C NMR (101 MHz, CDCl_3_) δ 173.4, 158.1, 154.2, 147.1, 144.6, 143.3, 140.8, 140.1, 137.8, 135.9, 133.9, 131.7, 127.0, 125.5, 122.1, 119.9, 118.0, 118.0, 117.9, 117.5, 115.0, 100.8, 97.4, 83.2, 80.3, 79.2, 76.9, 68.5, 68.3, 67.6, 66.9, 59.0, 55.5, 49.9, 49.2, 45.6, 41.2, 39.8, 39.1, 36.7, 35.7, 35.4, 34.0, 31.2, 28.0, 27.9, 27.4, 26.3, 22.5, 19.8, 18.6, 17.4, 14.4, 12.5, 11.8 ppm; ESI-MS *m*/*z*: [M + H]^+^ Calcd for C_55_H_73_N_2_O_11_^+^ 938; Found 938.

#### 4.5.3 Ivermectin–Cinchonine Conjugate **6**

109 mg, 21% yield (over two steps). Isolated as a white amorphous solid, a single spot by TLC. UV-active and strains green with PMA; ^1^H NMR (400 MHz, CDCl_3_) δ 8.84 (d, *J* = 4.5 Hz, 1H), 8.15–8.10 (m, 2H), 7.74–7.67 (m, 1H), 7.63–7.56 (m, 1H), 7.41 (d, *J* = 4.5 Hz, 1H), 6.47 (d, *J* = 6.1 Hz, 1H), 6.14–6.03 (m, 1H), 5.84–5.70 (m, 2H), 5.58 (dd, *J* = 13.7, 10.2 Hz, 1H), 5.40 (s, 1H), 5.35–5.25 (m, 1H), 5.13 (s, 1H), 5.10 (d, *J* = 4.4 Hz, 1H), 4.99 (dd, *J* = 9.6, 5.6 Hz, 1H), 4.92 (s, 1H), 4.64 (qd, *J* = 14.6, 1.9 Hz, 2H), 4.28 (s, 2H), 4.09 (dd, *J* = 14.3, 7.1 Hz, 1H), 3.97–3.90 (m, 1H), 3.61 (dd, *J* = 15.2, 9.7 Hz, 1H), 3.29–3.20 (m, 2H), 3.17 (d, *J* = 7.3 Hz, 1H), 2.99–2.87 (m, 2H), 2.87–2.75 (m, 1H), 2.75–2.54 (m, 3H), 2.26 (dd, *J* = 15.6, 7.5 Hz, 1H), 2.19–2.06 (m, 2H), 2.04–0.61 (m, 34H) ppm; ^13^C NMR (101 MHz, CDCl_3_) δ 173.4, 154.0, 149.6, 148.3, 145.1, 140.7, 140.0, 137.7, 135.9, 133.9, 130.3, 129.3, 127.0, 125.8, 125.4, 122.9, 119.9, 117.9, 117.8, 117.5, 115.0, 97.4, 83.1, 80.2, 79.1, 68.4, 68.2, 67.6, 66.9, 60.3, 59.5, 49.8, 49.1, 45.6, 41.2, 39.8, 39.0, 36.6, 35.7, 35.4, 34.0, 31.2, 27.9, 27.8, 27.4, 26.3, 22.8, 19.8, 18.6, 17.4, 14.4, 14.1, 12.5, 11.8 ppm; ESI-MS *m*/*z*: [M + H]^+^ Calcd for C_54_H_71_N_2_O_10_^+^ 908; Found 908.

#### 4.5.4 Ivermectin–Cinchonidine Conjugate **7**

88 mg, 17% yield (over two steps). Isolated as a white amorphous solid, a single spot by TLC. UV-active and strains green with PMA; ^1^H NMR (400 MHz, CDCl_3_) δ 8.89 (d, *J* = 4.5 Hz, 1H), 8.18 (d, *J* = 8.3 Hz, 1H), 8.13 (dd, *J* = 8.5, 0.9 Hz, 1H), 7.71 (ddd, *J* = 8.3, 6.9, 1.2 Hz, 1H), 7.59 (ddd, *J* = 8.3, 6.9, 1.2 Hz, 1H), 7.44 (d, *J* = 4.5 Hz, 1H), 6.37 (d, *J* = 6.5 Hz, 1H), 5.89–5.63 (m, 5H), 5.41 (s, 1H), 5.36–5.27 (m, 1H), 5.16–5.10 (m, 1H), 5.02–4.94 (m, 2H), 4.86 (s, 1H), 4.65 (qd, *J* = 14.5, 2.3 Hz, 2H), 4.28 (s, 2H), 4.10 (dd, *J* = 14.3, 7.1 Hz, 1H), 3.95 (d, *J* = 6.1 Hz, 1H), 3.71–3.62 (m, 1H), 3.34 (dd, *J* = 15.5, 7.3 Hz, 1H), 3.26 (dd, *J* = 4.5, 2.2 Hz, 1H), 3.19 (d, *J* = 7.6 Hz, 1H), 3.12 (s, 1H), 3.02 (dd, *J* = 13.8, 10.1 Hz, 1H), 2.70–2.53 (m, 4H), 2.33–2.22 (m, 3H), 2.08–0.69 (m, 33H) ppm; ^13^C NMR (101 MHz, CDCl_3_) δ 173.4, 154.1, 149.8, 148.4, 144.9, 141.4, 140.7, 137.7, 136.0, 134.3, 130.3, 129.3, 126.9, 125.7, 125.4, 123.0, 119.9, 118.1, 118.0, 117.7, 114.6, 97.4, 83.0, 80.2, 79.2, 77.9, 68.4, 68.2, 67.6, 67.0, 60.3, 59.7, 56.6, 45.6, 42.5, 41.2, 39.6, 39.0, 36.7, 35.7, 35.4, 34.2, 31.2, 28.0, 27.6, 27.4, 23.9, 19.8, 18.4, 17.4, 14.5, 14.1, 12.5, 11.8 ppm; ESI-MS *m*/*z*: [M + H]^+^ Calcd for C_54_H_71_N_2_O_10_^+^ 908; Found 908.

#### 4.5.5 Ivermectin–*N*-Deacetylthiocolchicine Conjugate **8**

79 mg, 14% yield (over two steps). Isolated as a yellow, amorphous solid, a single spot by TLC. UV-active and strains green with PMA; ^1^H NMR (400 MHz, CDCl_3_) δ 7.65 (s, 1H), 7.19 (dd, *J* = 38.4, 10.5 Hz, 2H), 7.05 (s, 1H), 6.54 (s, 1H), 5.68–5.57 (m, 1H), 5.47–5.35 (m, 2H), 5.27–5.17 (m, 1H), 5.03 (d, *J* = 9.7 Hz, 1H), 4.78–4.50 (m, 3H), 4.28 (d, *J* = 5.4 Hz, 1H), 3.97 (d, *J* = 6.1 Hz, 1H), 3.89 (d, *J* = 3.8 Hz, 3H), 3.75–3.60 (m, 2H), 3.50 (s, 2H), 3.28–3.20 (m, 1H), 2.62–0.69 (m, 48H) ppm; ^13^C NMR (101 MHz, CDCl_3_) δ 182.2, 173.0, 158.5, 155.0, 153.5, 151.0, 151.0, 141.4, 139.5, 138.0, 137.7, 137.3, 135.2, 134.7, 134.3, 129.1, 126.9, 125.6, 124.6, 120.4, 118.2, 117.3, 107.4, 97.4, 80.5, 79.3, 79.2, 76.0, 68.7, 68.2, 67.6, 67.1, 61.4, 61.3, 56.1, 53.4, 45.5, 41.3, 38.9, 37.5, 36.5, 35.7, 35.3, 34.0, 31.1, 30.0, 29.6, 28.1, 27.4, 19.9, 19.2, 17.4, 15.1, 14.4, 12.5, 12.4 ppm; ESI-MS *m*/*z*: [M + H]^+^ Calcd for C_55_H_72_NO_13_S^+^ 986; Found 987; [M + Na]^+^ Calcd for C_55_H_71_NNaO_13_S^+^ 1008; Found 1009.

### 4.6 Synthesis of IVR Aglycon-1H-imidazole-1-carboxylate (Compound **9**)

To a suspension of CDI (148 mg, 0.91 mmol, 2 equiv) in dry toluene (10 mL) was added dropwise a solution of compound **3** (320 mg, 0.46 mmol, 1 equiv) in dry toluene (5 mL). The resulting mixture was stirred at room temperature for 24 h. Thereafter, the reaction mixture was concentrated to dryness by using a rotary evaporator, dissolved in CH_2_Cl_2_, and extracted with brine. The organic layers were then concentrated under reduced pressure. The product was purified on silica gel using the CombiFlash system (0% → 10% EtOAc/CHCl_3_), yielding pure product **9** as an oil. After two evaporation steps with *n*-pentane to remove any remaining solvent, the oily product was fully converted to a yellow amorphous solid (215 mg, 59% yield). The spectroscopic data were in agreement with previously published data.^16^

### 4.7 General Procedure for the Preparation of Ivermectin Conjugates with Nucleosides and Metronidazole (Compounds **10–12**)

The appropriate conjugation partner (nucleoside analog or metronidazole, 1.5 equiv) was dissolved in DMF (20 mL), and after being heated to 90 °C, DBU (1.5 equiv) was added. After 30 min, a solution of **9** (1.0 g, 1.26 mmol, 1 equiv) in DMF (20 mL) was added dropwise to the reaction mixture, and stirring was continued for 24 h. Then, the reaction mixture was concentrated to dryness by using a rotary evaporator, diluted with CH_2_Cl_2_, and extracted with brine. The collected organic layers were subsequently concentrated *in vacuo*. Purification on silica gel using the CombiFlash system (0% → 100% EtOAc/CHCl_3_) gave the respective C5-protected bioconjugates as clear oils.

To remove the C5-protecting group, the individual hybrids were dissolved in methanol (10 mL), and *para*-toluenesulfonic acid hydrate (80 mg, 0.46 mmol, 1.3 equiv) was added. After 2 h, the organic solvent was evaporated, diluted with CH_2_Cl_2_, and extracted with an aqueous solution of sodium carbonate (0.1 M). Next, the collected organic layers were subsequently concentrated *in vacuo*. Purification on silica gel using the CombiFlash system (0% → 100% EtOAc/CHCl_3_) gave the pure products **10–12** as clear oils. After twice the evaporation to dryness with *n*-pentane, the oily products were completely converted into white amorphous solids.

#### 4.7.1 Ivermectin–Floxuridine Conjugate **10**

162 mg, 15% yield (over two steps). Isolated as a white amorphous solid, a single spot by TLC. UV-active and strains green with PMA; ^1^H NMR (400 MHz, CDCl_3_) δ 9.83 (s, 1H), 7.73 (d, *J* = 6.1 Hz, 1H), 6.27 (t, *J* = 6.0 Hz, 1H), 6.18–6.10 (m, 2H), 5.70 (ddd, *J* = 24.3, 14.9, 10.1 Hz, 2H), 5.30 (td, *J* = 10.6, 5.5 Hz, 1H), 5.09 (d, *J* = 9.8 Hz, 1H), 4.98 (s, 1H), 4.62–4.33 (m, 5H), 4.21 (d, *J* = 3.3 Hz, 1H), 4.05 (d, *J* = 1.6 Hz, 1H), 3.73–3.64 (m, 1H), 3.59 (d, *J* = 9.1 Hz, 1H), 3.17 (d, *J* = 8.1 Hz, 1H), 2.66–2.58 (m, 2H), 2.55–2.46 (m, 2H), 2.37–0.57 (m, 35H) ppm; ^13^C NMR (101 MHz, CDCl_3_) δ 168.3, 156.9, 156.6, 154.6, 148.9, 141.9, 140.0, 139.5, 138.6, 135.7, 133.5, 129.6, 126.5, 122.4, 118.1, 97.3, 85.8, 84.5, 83.6, 83.0, 78.5, 77.2, 72.1, 71.0, 68.7, 68.1, 66.9, 40.5, 39.1, 36.7, 35.8, 35.5, 34.4, 33.1, 31.2, 29.7, 28.0, 27.2, 18.7, 17.4, 16.8, 14.5, 12.5, 11.7 ppm; ESI-MS *m*/*z*: [M + Na]^+^ Calcd for C_44_H_59_FN_2_NaO_14_^+^ 881; Found 881.

#### 4.7.2 Ivermectin–Azidothymidine Conjugate **11**

321 mg, 29% yield (over two steps). Isolated as a white amorphous solid, a single spot by TLC. UV-active and strains green with PMA; ^1^H NMR (400 MHz, CDCl_3_) δ 9.55 (s, 1H), 7.27 (d, *J* = 0.8 Hz, 1H), 6.15–6.05 (m, 2H), 5.67 (ddd, *J* = 24.7, 14.8, 10.6 Hz, 3H), 5.29–5.20 (m, 1H), 5.05 (d, *J* = 8.2 Hz, 1H), 4.96 (s, 1H), 4.84 (s, 1H), 4.53 (dd, *J* = 30.0, 14.4 Hz, 2H), 4.39 (ddd, *J* = 15.4, 12.0, 4.2 Hz, 2H), 4.22 (dd, *J* = 12.3, 5.2 Hz, 1H), 4.10–4.01 (m, 2H), 3.68–3.59 (m, 1H), 3.58–3.52 (m, 1H), 3.13 (d, *J* = 7.7 Hz, 1H), 2.79 (d, *J* = 6.5 Hz, 1H), 2.64–2.55 (m, 1H), 2.53–2.41 (m, 2H), 2.41–2.30 (m, 1H), 2.30–2.15 (m, 2H), 2.04–0.55 (m, 33H) ppm; ^13^C NMR (101 MHz, _CDCl3_) δ 168.3, 163.6, 154.3, 150.2, 140.1, 138.8, 135.4, 133.6, 129.5, 126.5, 122.2, 117.8, 111.3, 97.2, 85.6, 83.6, 83.0, 81.6, 78.3, 77.1, 71.9, 68.5, 68.0, 66.8, 66.7, 60.4, 40.3, 38.9, 37.4, 36.6, 35.7, 35.4, 34.4, 33.1, 31.1, 29.6, 27.8, 27.2, 18.6, 17.3, 16.8, 14.4, 12.6, 12.4, 11.6 ppm; ESI-MS *m*/*z*: [M + Na]^+^ Calcd for C_45_H_61_N_5_NaO_13_^+^ 902; Found 902.

#### 4.7.3 Ivermectin–Metronidazole Conjugate **12**

296 mg, 30% yield (over two steps). Isolated as a white amorphous solid, a single spot by TLC. UV-active and strains green with PMA; ^1^H NMR (401 MHz, CDCl_3_) δ 7.88 (s, 1H), 6.07 (dd, *J* = 7.2, 2.2 Hz, 2H), 5.61 (ddd, *J* = 24.8, 15.0, 10.3 Hz, 2H), 5.25–5.16 (m, 1H), 4.91 (d, *J* = 10.8 Hz, 1H), 4.81 (s, 1H), 4.79 (d, *J* = 15.5 Hz, 1H), 4.59–4.49 (m, 4H), 4.45 (dd, *J* = 12.2, 2.0 Hz, 2H), 4.37 (ddd, *J* = 11.3, 10.5, 5.4 Hz, 2H), 3.95 (d, *J* = 2.1 Hz, 1H), 3.64–3.56 (m, 1H), 3.50 (dd, *J* = 9.4, 2.0 Hz, 1H), 3.38 (s, 1H), 3.11 (d, *J* = 7.7 Hz, 1H), 2.57–0.51 (m, 35H) ppm; ^13^C NMR (101 MHz, CDCl_3_) δ 168.5, 154.2, 150.8, 140.3, 138.6, 135.6, 133.5, 133.0, 129.6, 126.4, 122.2, 117.9, 97.2, 83.6, 82.9, 78.4, 77.2, 72.0, 68.6, 68.0, 66.8, 65.7, 45.2, 40.4, 38.9, 36.7, 35.8, 35.4, 34.4, 33.1, 31.2, 27.9, 27.3, 18.5, 17.3, 16.8, 14.4, 14.3, 12.4, 11.6 ppm; ESI-MS *m*/*z*: [M + Na]^+^ Calcd for C_41_H_57_N_3_NaO_12_^+^ 806; Found 806.

### 4.8 Synthesis of Betulinic Acid Propargyl Ester

A mixture of betulinic acid (2.0 g, 4.39 mmol, 1.0 equiv), DBU (1.0 g, 6.58 mmol, 1.5 equiv), and propargyl bromide (1.57 g, 13.16 mmol, 3.0 equiv) in anhydrous toluene (50 mL) was heated at 90 °C for 24 h. Subsequently, the mixture was concentrated under reduced pressure. Purification on silica gel using the CombiFlash system (0 → 50% EtOAc/*n*-hexane) yielded the pure product of the reaction (62% yield) as a clear oil. The oil was then diluted in *n*-pentane and evaporated to dryness three times to yield an amorphous solid. The spectroscopic data were in agreement with previously published data.^36^

### 4.9 Synthesis of Artesunate Propargyl Ester

To a stirred solution of artesunate (1.0 g, 2.60 mmol, 1.0 equiv) in anhydrous CH_2_Cl_2_ (40 mL) were added DCC (1.07 g, 5.20 mmol, 2.0 equiv) and a catalytic amount of DMAP. After 30 min, propargyl alcohol (730 mg, 13.02 mmol, 5 equiv) was added dropwise, and stirring was continued for 24 h. The formation of a white precipitate of DCU was observed. Afterward, the white precipitate was filtered off, and the reaction mixture was concentrated to dryness using a rotary evaporator. The product was then purified on silica gel using the CombiFlash system (0% → 100% EtOAc/CHCl_3_), yielding the pure product of the reaction (47% yield) as a clear oil. The oil was then diluted in *n*-pentane and evaporated to dryness three times to yield an amorphous solid. The spectroscopic data were in agreement with previously published data.^37^

### 4.10 Synthesis of Ivermectin C13-Chloroacetyl Ester (Compound **13**)

Precursor **3** (1.0 g, 1.43 mmol, 1.0 equiv) was dissolved in anhydrous CH_2_Cl_2_ (30 mL). The solution was then cooled in an ice bath, and pyridine (564 mg, 7.13 mmol, 5.0 equiv) was added. Next, chloroacetyl chloride (322 mg, 2.85 mmol, 2.0 equiv) was added dropwise to the reaction mixture. The solution turned brown, and stirring was continued for 24 h. Thereafter, the reaction mixture was concentrated to dryness using a rotary evaporator, diluted with CH_2_Cl_2_, and extracted with an aqueous solution of sulfuric acid (pH = 1) and then with water. The collected organic layers were subsequently concentrated *in vacuo*. Purification on silica gel using the CombiFlash system (0% → 10% EtOAc/CHCl_3_) gave the respective product of the reaction (72% yield) as a clear oil. The oil was then diluted in *n*-pentane and evaporated to dryness three times to yield an amorphous solid. The spectroscopic data were in agreement with the previously published data.^15^

### 4.11 Synthesis of Ivermectin C13-Azide Precursor (Compound **14**)

Ivermectin C13-chloroacetyl ester **13** (400 mg, 0.51 mmol, 1.0 equiv) was dissolved in DMF (10 mL). Sodium azide (67 mg, 1.02 mmol, 2.0 equiv) was then added in one portion, the mixture was heated to 60 °C, and stirring was continued for 24 h. Afterward, the reaction mixture was diluted with a large amount of water, and extracted several times with methylene chloride. This step can be hazardous due to the potential for an explosion resulting from the reaction between sodium azide and the chlorinated solvent, so it is essential to follow the sequence of operations precisely. The collected organic layers were subsequently concentrated *in vacuo*. This raw mixture was used directly in the next step.

### 4.12 Synthesis of Ivermectin-Betulinic Acid Conjugate (Compound **15**)

Under a nitrogen atmosphere, a solution of **14** (∼1.0 equiv) in anhydrous CH_3_CN was prepared. Betulinic acid propargyl ester (157 mg, 0.32 mmol, 1.0 equiv) and DIPEA (123 mg, 0.95 mmol, 3.0 equiv) were then added, followed by the addition of catalytic CuI (6.05 mg, 0.03 mmol, 0.1 equiv) in one portion. The reaction mixture was stirred at room temperature for 24 h. After complete consumption of the propargyl partner (monitored by TLC and ESI-MS), the organic solvent was removed by using a rotary evaporator. The oily residue was dissolved in a small amount of CH_2_Cl_2_ and extracted several times with 10% aqueous EDTA solution. The organic phases were separated and concentrated under reduced pressure. The product was purified by silica gel chromatography using the CombiFlash system (0 → 100% EtOAc/CHCl_3_), yielding the pure product of the click reaction as a clear oil. The oil was diluted in *n*-pentane and evaporated to dryness three times to obtain the amorphous solid.

To remove the C5-protecting group, the hybrid was dissolved in methanol (10 mL), and *para*-toluenesulfonic acid hydrate (80 mg, 0.46 mmol, 1.3 equiv) was added. After 2 h, the organic solvent was evaporated, diluted with CH_2_Cl_2_, and extracted with an aqueous solution of sodium carbonate (0.1 M). Next, the collected organic layers were subsequently concentrated *in vacuo*. Purification on silica gel using the CombiFlash system (0% → 100% EtOAc/CHCl_3_) gave the pure product **15** as s clear oil. After twice evaporation to dryness with *n*-pentane, the oily product was completely converted into a white amorphous solid.

#### 4.12.1 Ivermectin–Betulinic Acid Conjugate **15**

122 mg, 33% yield (over two steps). Isolated as a white amorphous solid, a single spot by TLC. UV-active and strains green with PMA; ^1^H NMR (401 MHz, CD_2_Cl_2_) δ 7.78 (s, 1H), 6.17 (d, *J* = 2.0 Hz, 1H), 6.12 (dt, *J* = 10.8, 2.4 Hz, 1H), 5.81 (ddd, *J* = 20.3, 10.5, 6.7 Hz, 1H), 5.72–5.62 (m, 1H), 5.32 (m, 1H), 5.30–5.25 (m, 3H), 5.22 (dd, *J* = 9.7, 3.6 Hz, 3H), 5.06–5.00 (m, 1H), 4.75 (s, 1H), 4.72 (d, *J* = 2.3 Hz, 1H), 4.58 (dd, *J* = 2.4, 1.4 Hz, 1H), 4.58–4.45 (m, 2H), 3.97 (d, *J* = 2.2 Hz, 1H), 3.76–3.67 (m, 1H), 3.55–3.47 (m, 1H), 3.24 (dd, *J* = 9.9, 5.1 Hz, 1H), 3.13 (dd, *J* = 12.1, 4.0 Hz, 1H), 3.02–2.95 (m, 1H), 2.73–2.62 (m, 1H), 2.53–0.62 (m, 76H) ppm; ^13^C NMR (101 MHz, CD_2_Cl_2_) δ 176.3, 169.2, 166.2, 151.3, 144.2, 141.2, 139.3, 136.2, 134.0, 130.4, 127.2, 125.8, 122.7, 118.7, 109.9, 97.9, 83.7, 81.6, 79.2, 79.0, 77.7, 72.7, 69.3, 68.7, 67.5, 57.6, 57.0, 55.8, 51.2, 51.1, 50.0, 47.6, 42.9, 41.3, 41.0, 39.5, 39.3, 39.3, 38.8, 37.7, 37.4, 36.4, 36.1, 35.0, 34.8, 33.9, 32.5, 31.8, 31.1, 30.1, 28.5, 28.3, 28.0, 28.0, 26.1, 21.4, 19.6, 19.2, 18.8, 17.8, 17.2, 16.5, 16.1, 15.8, 15.0, 14.9, 13.0, 12.1 ppm; ESI-MS *m*/*z*: [M + Na]^+^ Calcd for C_69_H_101_N_3_NaO_12_^+^ 1187; Found 1188.

### 4.13 Synthesis of Ivermectin-Artesunate Conjugate (Compound **16**)

To remove the C5-protecting group, azide **14** was dissolved in methanol (10 mL), and *para*-toluenesulfonic acid hydrate (80 mg, 0.46 mmol, 1.3 equiv) was added. After 2 h, the organic solvent was evaporated, diluted with CH_2_Cl_2_, and extracted with an aqueous solution of sodium carbonate (0.1 M). Next, the collected organic layers were subsequently concentrated *in vacuo*. This raw mixture was used directly in the next step.

Under a nitrogen atmosphere, a solution of deprotected azide (∼1.0 equiv) in anhydrous CH_3_CN was prepared. Artesunate propargyl ester (135 mg, 0.32 mmol, 1.0 equiv) and DIPEA (123 mg, 0.95 mmol, 3.0 equiv) were then added, followed by the addition of catalytic CuI (6.05 mg, 0.03 mmol, 0.1 equiv) in one portion. The reaction mixture was stirred at room temperature for 24 h. After complete consumption of the propargyl partner (monitored by TLC and ESI-MS), the organic solvent was removed using a rotary evaporator. The oily residue was dissolved in a small amount of CH_2_Cl_2_ and extracted several times with 10% aqueous EDTA solution. The organic phases were separated and concentrated under reduced pressure. The product was purified by silica gel chromatography using the CombiFlash system (0 → 100% EtOAc/CHCl_3_), yielding the pure product **16** of the click reaction as a clear oil. The oil was diluted in *n*-pentane and evaporated to dryness three times to obtain the amorphous solid.

#### 4.13.1 Ivermectin–Artesunate Conjugate **16**

220 mg, 63% yield (yield of one step–click reaction). Isolated as a white amorphous solid, a single spot by TLC. UV-active and strains green with PMA; ^1^H NMR (401 MHz, CDCl_3_) δ 7.80 (s, 1H), 6.18–6.13 (m, 2H), 5.75 (dt, *J* = 11.0, 8.0 Hz, 2H), 5.62 (dd, *J* = 15.0, 10.0 Hz, 1H), 5.42 (s, 1H), 5.35–5.26 (m, 5H), 5.21 (s, 1H), 4.94 (d, *J* = 11.2 Hz, 1H), 4.83 (s, 1H), 4.53 (qd, *J* = 14.1, 2.2 Hz, 2H), 4.04 (d, *J* = 2.2 Hz, 1H), 3.74–3.65 (m, 1H), 3.61–3.55 (m, 1H), 3.20 (d, *J* = 7.8 Hz, 1H), 2.78–0.61 (m, 60H) ppm; ^13^C NMR (101 MHz, CDCl^3^) δ 171.8, 170.9, 168.6, 165.3, 143.2, 140.5, 138.7, 135.5, 133.4, 129.6, 126.5, 125.2, 122.2, 118.1, 104.4, 97.3, 92.1, 91.4, 82.8, 81.0, 80.0, 78.4, 72.1, 68.7, 68.1, 66.8, 57.9, 51.5, 50.5, 45.1, 40.4, 38.8, 37.2, 36.7, 36.1, 35.8, 35.4, 34.4, 34.0, 33.2, 31.7, 31.2, 29.6, 29.1, 28.7, 27.9, 27.4, 25.8, 24.5, 21.9, 20.1, 18.8, 17.4, 16.8, 14.5, 12.6, 11.9, 11.7 ppm; ESI-MS *m*/*z*: [M + Na]^+^ Calcd for C_58_H_81_N_3_NaO_17_^+^ 1115; Found 1114.

### 4.14 In Vitro Biological Studies

#### 4.14.1 Trypanocidal and Cytotoxic Assays

The 427–221a clone of bloodstream forms of *T. b. brucei* ^38^ and human promyelocytic leukemia HL-60 cells^39^ were used for evaluating the trypanocidal and cytotoxic activity of test compounds. Trypanosomes and HL-cells were seeded in 96-well plates at cell densities of 1 × 10^–4^ and 5 × 10^–4^ mL^–1^, respectively, in 200 μL of Baltz medium^40^ supplemented with 16.7% bovine serum. Test compounds were assayed at 10-fold dilutions ranging from 100 μM to 1 nM in the presence of 0.9% DMSO. Controls were grown in the culture medium containing only 0.9% DMSO. The assays were cultured in an incubator at 37 °C in a humidified atmosphere containing 5% CO_2_. After 24 h incubation, 20 μL of 0.5 mM resazurin (prepared in sterile PBS) was added, and the cultures were grown for another 48 h. Then, the proliferation of the cells was determined by measuring the absorbance at 570 nm (test wavelength) and 630 nm (reference wavelength) using a microplate reader. The half-maximal growth inhibition (GI_50_) values, i.e., the concentration of a test compound that reduces the growth rate of cells by 50% compared to the growth rate of the control cells, were calculated using the method described by Huber and Koella.^41^

#### 4.14.2 Human Cell Lines

The human cancer cell lines PC3 (metastatic prostate cancer), MDA-MB-231 (breast cancer), A549 (lung cancer), HCT-116 (colon carcinoma), and the immortalized human keratinocyte line HaCaT were obtained from the repository of the Medical University of Warsaw. PC3 cells were cultured in RPMI (Biowest SAS, France) and HCT-116 cells in MEM (Thermo Scientific, USA), while A549, MDA-MB-231, and HaCaT cells were maintained in DMEM High Glucose (Biowest SAS, France). The culture media were enriched with 10% fetal bovine serum (FBS, Sigma-Aldrich, St. Louis, MO, USA), 20 mM HEPES (Biowest, Nuaillé, France), and antibiotics (100 U/mL penicillin and 100 μg/mL streptomycin) obtained from Gibco (Grand Island, NY, USA). The cells were maintained in a humidified incubator at 37 °C with 5% CO_2_ until they reached 80–90% confluence, after which they were used for further experiments.

#### 4.14.3 Cytotoxicity

To assess the cytotoxicity, the cells (0.5 × 10^4^/well) were plated in 96-well plates and allowed to adhere for 24 h. The MTT (3-(4,5-dimethylthiazol-2-yl)-2,5-diphenyltetrazolium bromide) assay was used to evaluate the cytotoxic effects of **IVR** bioconjugates on all tested cell lines. After 24 h incubation, cells were exposed to varying concentrations (1 to 100 μM) of the test compounds for 72 h. Following the treatment, the medium was discarded, and a 0.5 mg mL^–1^ MTT solution was added to each well and incubation was continued for another 4 h. Mitochondrial enzyme activity facilitated the formation of formazan crystals, which were subsequently dissolved in DMSO-isopropanol (1:1, v/v), producing a violet-colored solution. A 100 μL aliquot of this solution was transferred to the well, and absorbance was recorded at 570 nm using a MultiscanGo spectrophotometer (ThermoFisher Scientific, Carlsbad, CA, USA).

Cytotoxicity was quantified by comparing the absorbance of the treated cells to that of untreated controls using the formula: [A]/[B] × 100, where [A] represents the absorbance of the treated sample and [B] corresponds to the absorbance of the control sample. A decrease in MTT levels reflects a decrease in cell viability. IC_50_ values, the concentration required to inhibit 50% of cell viability, were calculated using GraphPad Prism 8.0.1 software.

## Supplementary Material



## Data Availability

The data underlying this study are available in the published article and its Supporting Information.

## Notes

General procedures, as well as NMR and ESI-MS spectra of the reported compounds (PDF)

## Abbreviations

A549: lung cancer cell line
CDI: 1,1’-carbonyldiimidazole
CuAAC: copper-catalyzed azide–alkyne cycloaddition
DBU: 1,8-diazabicyklo­[5.4.0]­undek-7-en
DCC: *N*,*N*′-dicyclohexylcarbodiimide)
DIPEA: *N*,*N*-diisopropylethylamine
DMAP: 4-dimethylaminopyridine
DMF: *N*,*N*-dimethylformamide
DNA: DNA
ESI-MS: electrospray ionization mass spectrometry
GI50: half-maximal growth inhibition
HaCaT: human keratinocytes
HCT-116: primary colon cancer
HL-60: promyelocytic leukemia cell line
IC50: half-maximal inhibitory concentration
IVR: ivermectin
MDA-MB-231: triple-negative breast adenocarcinoma
MDR: multidrug resistance
MTT: 3-(4,5-dimethylthiazol-2-yl)-2,5-diphenyltetrazolium bromide
NMR: nuclear magnetic resonance
PC3: metastatic prostate cancer
pTsOH: para-toluenesulfonic acid
pyr: pyridine
ROS: reactive-oxygen species
rt: room temperature
SI: selectivity index
TBDMSCl: *tert*-butyldimethylsilyl chloride
TEA: triethylamine
THF: tetryhydrofuran
WHO: World Health Organization
