# Supplementary material for: Antiproliferative and Trypanocidal Activity of Ivermectin Bioconjugates
Source: ACS Omega. 2025 Jun 18;10(25):27380–92. doi: 10.1021/acsomega.5c02998 (PMC12223813; doi:10.1021/acsomega.5c02998)
Supplement: Supplementary file 1 [file ao5c02998_si_001.pdf]

Antiproliferative and trypanocidal activity of ivermectin bioconjugates

Michał Sulik [a], Dagmara Otto-Ślusarczyk [b], Dietmar Steverding [c], Marta Struga [b], Adam Huczyński [a,*]

[a] *Department of Medical Chemistry, Faculty of Chemistry, Adam Mickiewicz University, Uniwersytetu Poznańskiego 8, 61–614 Poznań, Poland*

[b] *Chair and Department of Biochemistry, Faculty of Medicine, Medical University of Warsaw, Banacha 1, 02–097, Warsaw, Poland*

[c] *Bob Champion Research & Education Building, Norwich Medical School, University of East Anglia, Norwich, U.K.*

Supplementary material

Index

General procedures S2

Spectroscopic and spectrometric analysis of newly synthesized compounds................................S3

*E-mail: adhucz@amu.edu.pl (A. Huczyński)

General procedures

All reagents were purchased from two sources – Merck or Trimen Chemicals S.A., and used without further purification. $CDCl_3$ and CD_2Cl_2 spectral grade were stored over 3Å molecular sieves for several days. Reaction mixtures were stirred using Teflon-coated magnetic stir bars and were monitored by thin layer chromatography (TLC) using aluminum-backed plates (Merck 60 F_{254}). TLC plates were visualized by UV-light (254 nm), followed by treatment with phosphomolybdic acid (PMA) (5% in absolute ethanol) and gentle heating. Products of the reactions were purified using CombiFlash® Rf+ Lumen Flash Chromatography System (Teledyne Isco) with integrated ELS and UV detectors. All solvents used in flash chromatography were of HPLC grade (Merck) and were used as received. Solvents were removed using a rotary evaporator.

NMR spectra were recorded on a Varian 400 (^{1}H NMR at 400 MHz and ^{13}C NMR at 101 MHz) magnetic resonance spectrometer. ^{1}H NMR spectra are reported in chemical shifts downfield from TMS using the respective residual solvent peak as internal standard ($CDCl_3$ δ 7.26 ppm; CD_2Cl_2 δ 5.32 ppm). ^{1}H NMR spectra are reported as follows: chemical shift (δ, ppm), multiplicity (s = singlet, d = doublet, t = triplet, q = quartet, dd = doublet of doublets, dt = doublet of triplets, td = triplet of doublets, qd = quartet of doublets, ddd = doublet of doublets of doublets, m = multiplet), coupling constant(s) in Hz, and integration. Significant peaks are reported within the overlapping ~2.50–0.50 ppm region of the ^{1}H NMR spectra. ^{13}C NMR spectra are reported in chemical shifts downfield from TMS using the respective residual solvent peak as internal standard ($CDCl_3$ δ 77.16 ppm; CD_2Cl_2 δ 53.84 ppm). Line broadening parameters were 0.5 or 1.0 Hz, while the error of chemical shift value was 0.1 ppm.

The electrospray ionization (ESI) mass spectra were recorded on a Waters/Micromass ZQ mass spectrometer (Waters Alliance) equipped with a Harvard syringe pump. The samples were prepared in dry acetonitrile, and were infused into the ESI source using a Harvard pump at a flow rate of 20 ml/min. The ESI source potentials were: capillary 3 kV, lens 0.5 kV, and extractor 4 V. The standard ESI mass spectra were recorded at the cone voltages of 10 V. The source temperature was 120 °C and the desolvation temperature was 300°C. Nitrogen was used as the nebulizing and desolvation gas at flow-rates of 100 dm^3/h. Mass spectra were acquired in the positive ion detection mode with unit mass resolution at a step of 1 m/z unit. The mass range for ESI experiments was from m/z = 600 to m/z = 1200 or from m/z = 600 to m/z = 1400.

Spectroscopic and spectrometric analysis of newly synthesized compounds

List of spectra

Figure S1. The $^{13}C\{^{1}H\}$ NMR (101 MHz) spectrum of **4** in chloroform-d...S4

Figure S2. The ^{1}H NMR (400 MHz) spectrum of **4** in chloroform-d..S4

Figure S3. The $^{13}C\{^{1}H\}$ NMR (101 MHz) spectrum of **5** in chloroform-d...S5

Figure S4. The ^{1}H NMR (400 MHz) spectrum of **5** in chloroform-d..S5

Figure S5. The $^{13}C\{^{1}H\}$ NMR (101 MHz) spectrum of **6** in chloroform-d...S6

Figure S6. The ^{1}H NMR (400 MHz) spectrum of **6** in chloroform-d..S6

Figure S7. The $^{13}C\{^{1}H\}$ NMR (101 MHz) spectrum of **7** in chloroform-d...S7

Figure S8. The ^{1}H NMR (400 MHz) spectrum of **7** in chloroform-d..S7

Figure S9. The $^{13}C\{^{1}H\}$ NMR (101 MHz) spectrum of **8** in chloroform-d...S8

Figure S10. The ^{1}H NMR (400 MHz) spectrum of **8** in chloroform-d..S8

Figure S11. The $^{13}C\{^{1}H\}$ NMR (101 MHz) spectrum of **10** in chloroform-d.....................................S9

Figure S12. The ^{1}H NMR (400 MHz) spectrum of **10** in chloroform-d..S9

Figure S13. The $^{13}C\{^{1}H\}$ NMR (101 MHz) spectrum of **11** in chloroform-d...................................S10

Figure S14. The ^{1}H NMR (400 MHz) spectrum of **11** in chloroform-d..S10

Figure S15. The $^{13}C\{^{1}H\}$ NMR (101 MHz) spectrum of **12** in chloroform-d...................................S11

Figure S16. The ^{1}H NMR (400 MHz) spectrum of **12** in chloroform-d..S11

Figure S17. The $^{13}C\{^{1}H\}$ NMR (101 MHz) spectrum of **15** in methylene chloride-d2.....................S12

Figure S18. The ^{1}H NMR (400 MHz) spectrum of **15** in methylene chloride-d2..............................S12

Figure S19. The $^{13}C\{^{1}H\}$ NMR (101 MHz) spectrum of **16** in chloroform-d...................................S13

Figure S20. The ^{1}H NMR (400 MHz) spectrum of **16** in chloroform-d..S13

Figure S21. The ESI mass spectra of a mixture of **4** (a), **5** (b), **6** (c), and **7** (d), with $NaClO_4$ at cv = 10 V. S14

Figure S22. The ESI mass spectra of a mixture of **8** (a), **10** (b), **11** (c), and **12** (d), with $NaClO_4$ at cv = 10 V. S15

Figure S23. The ESI mass spectra of a mixture of **15** (a), and **16** (b) with $NaClO_4$ at cv = 10 V.......S16

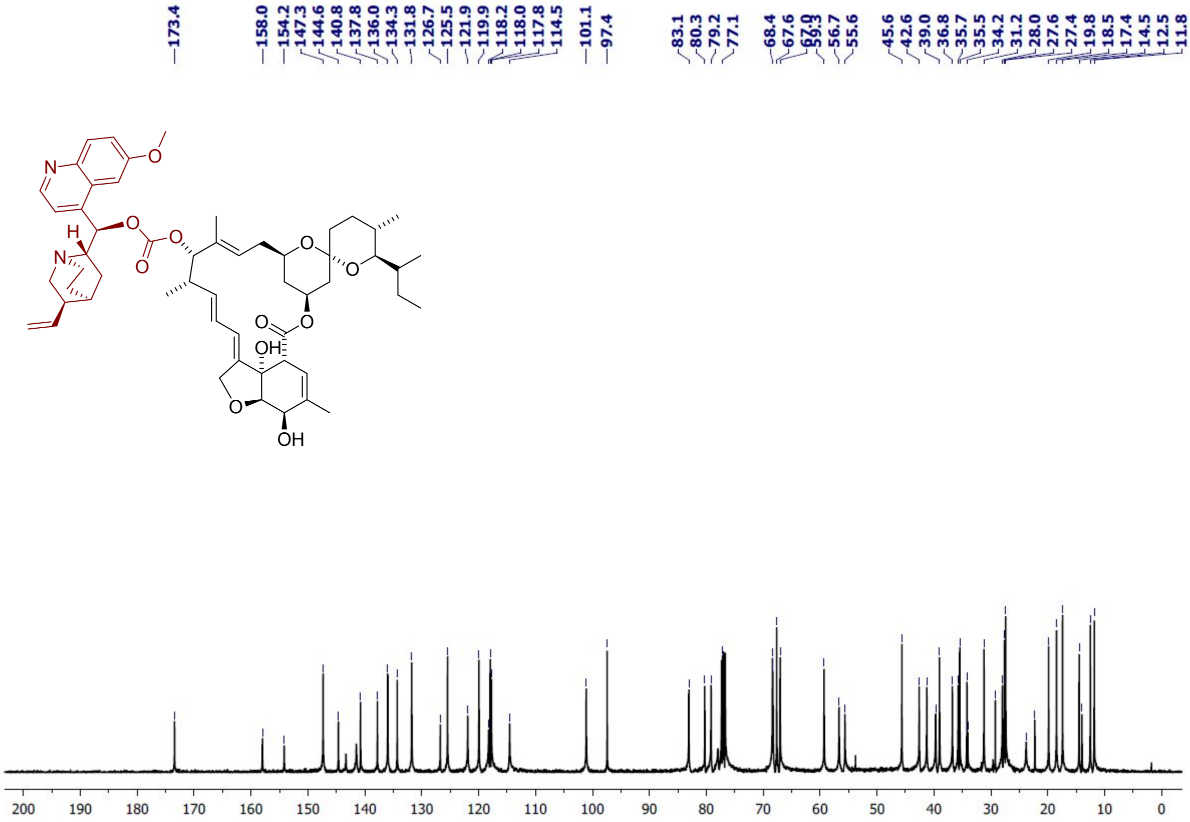

Figure S1. The $^{13}C\{^{1}H\}$ NMR (101 MHz) spectrum of **4** in chloroform-d.

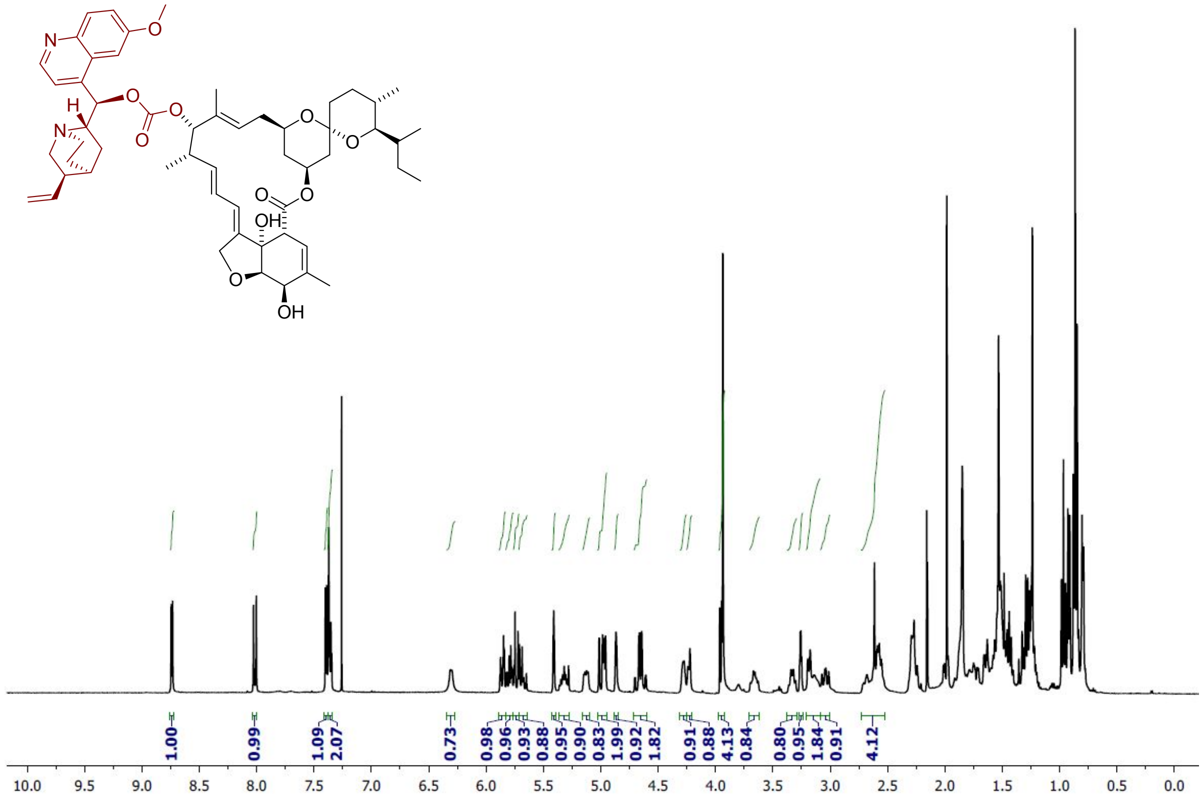

Figure S2. The ^{1}H NMR (400 MHz) spectrum of **4** in chloroform-d.

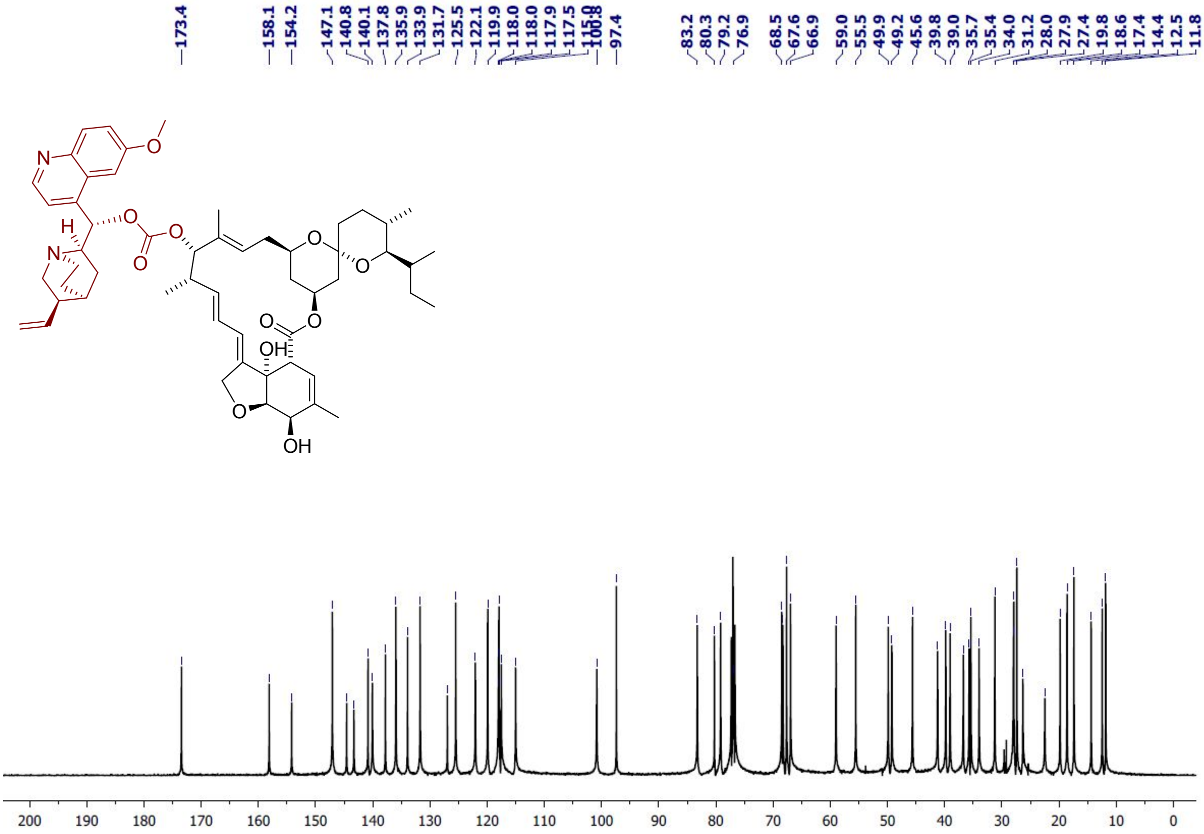

Figure S3. The $^{13}C\{^1H\}$ NMR (101 MHz) spectrum of **5** in chloroform-d.

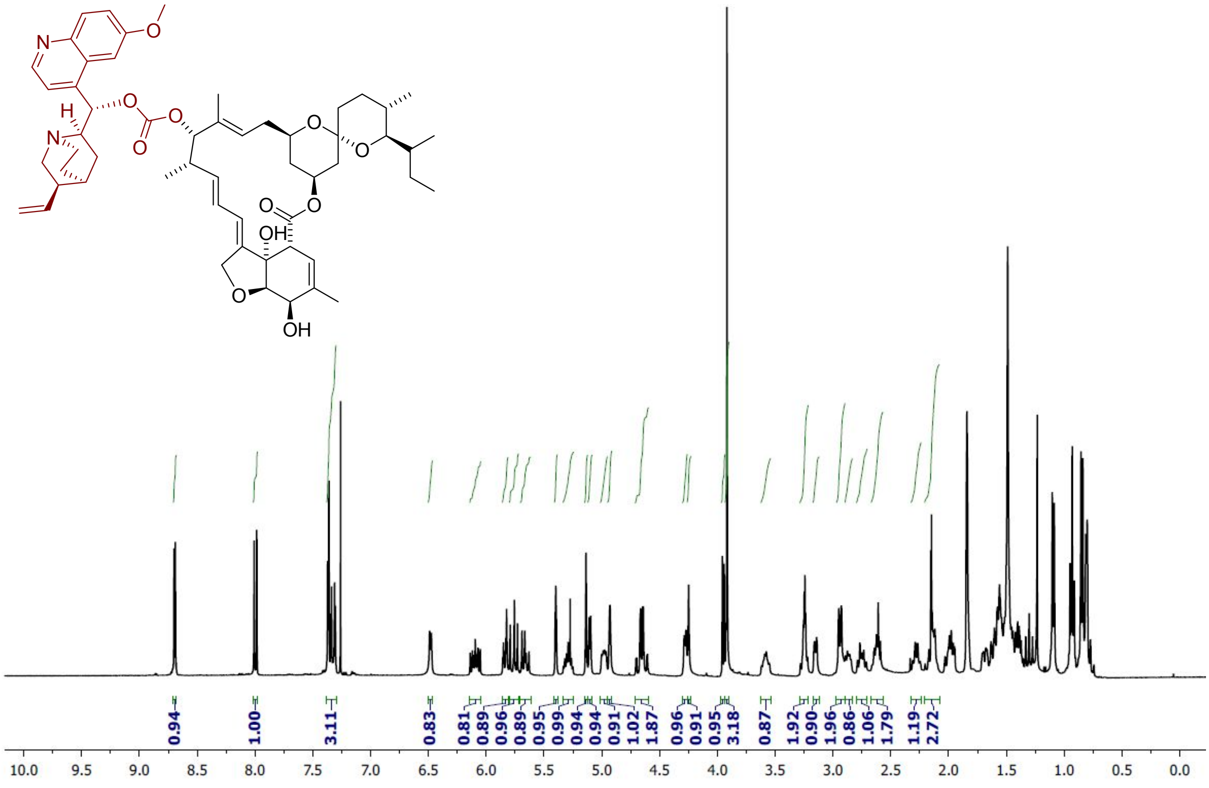

Figure S4. The ^1H NMR (400 MHz) spectrum of **5** in chloroform-d.

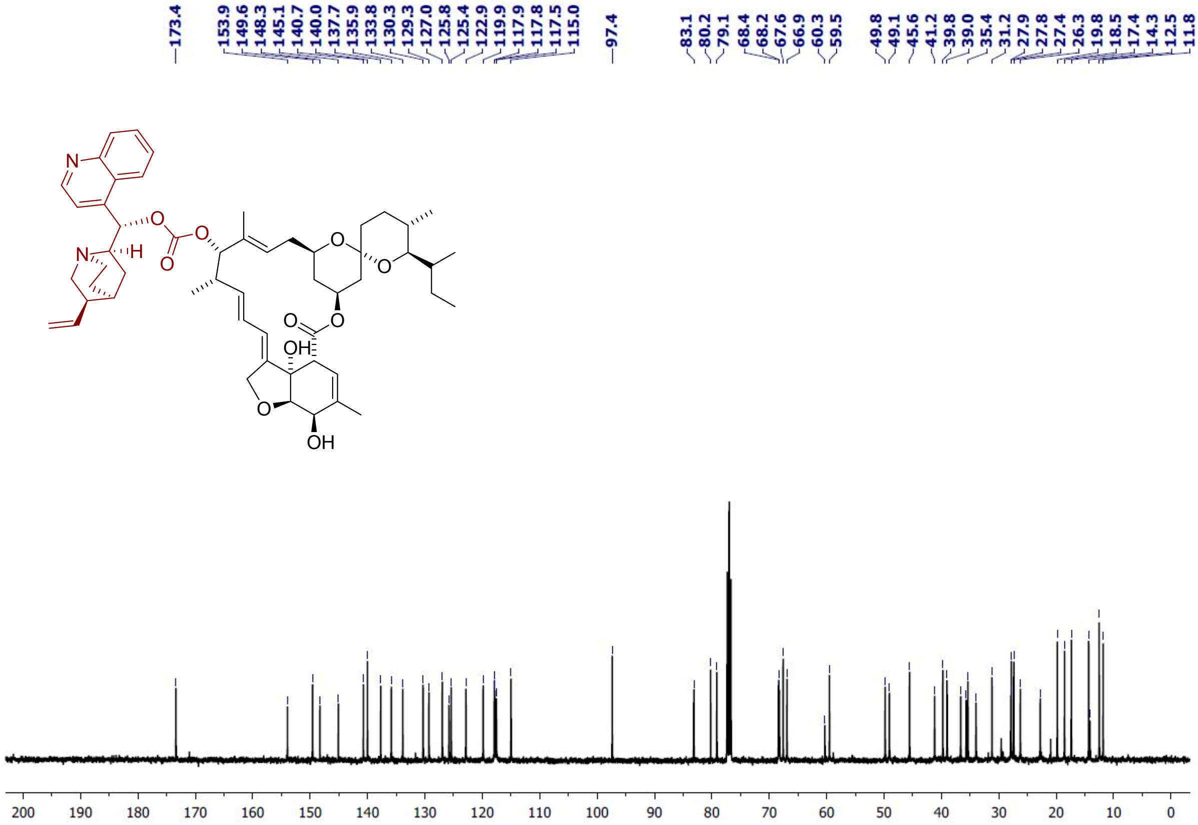

Figure S5. The $^{13}C\{^{1}H\}$ NMR (101 MHz) spectrum of **6** in chloroform-d.

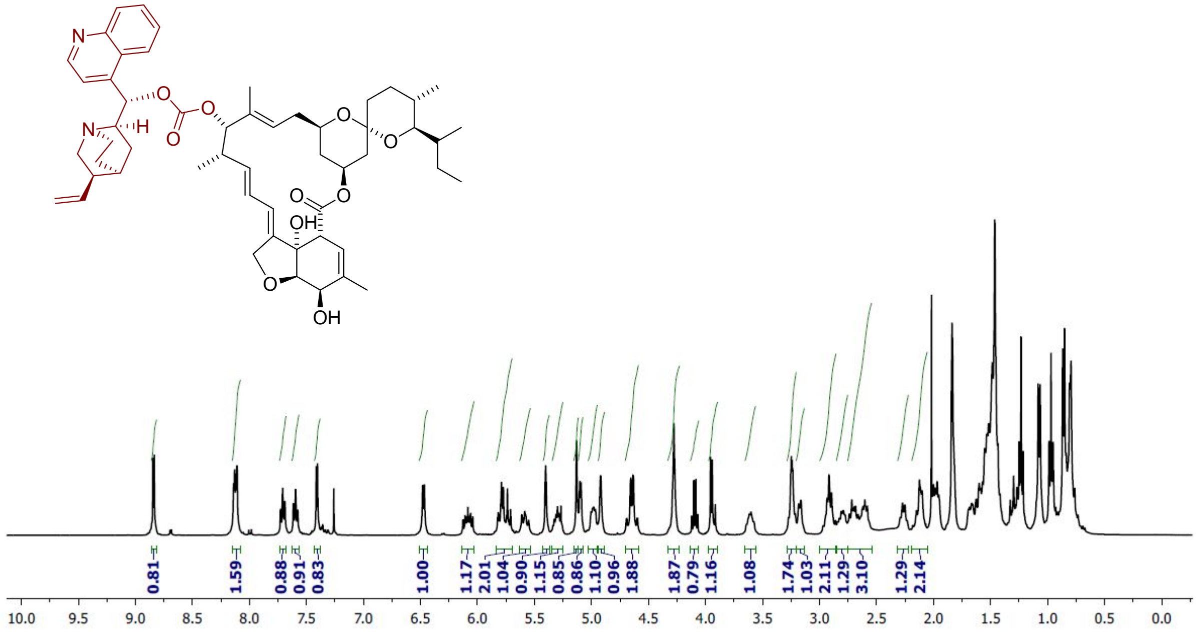

Figure S6. The ^{1}H NMR (400 MHz) spectrum of **6** in chloroform-d.

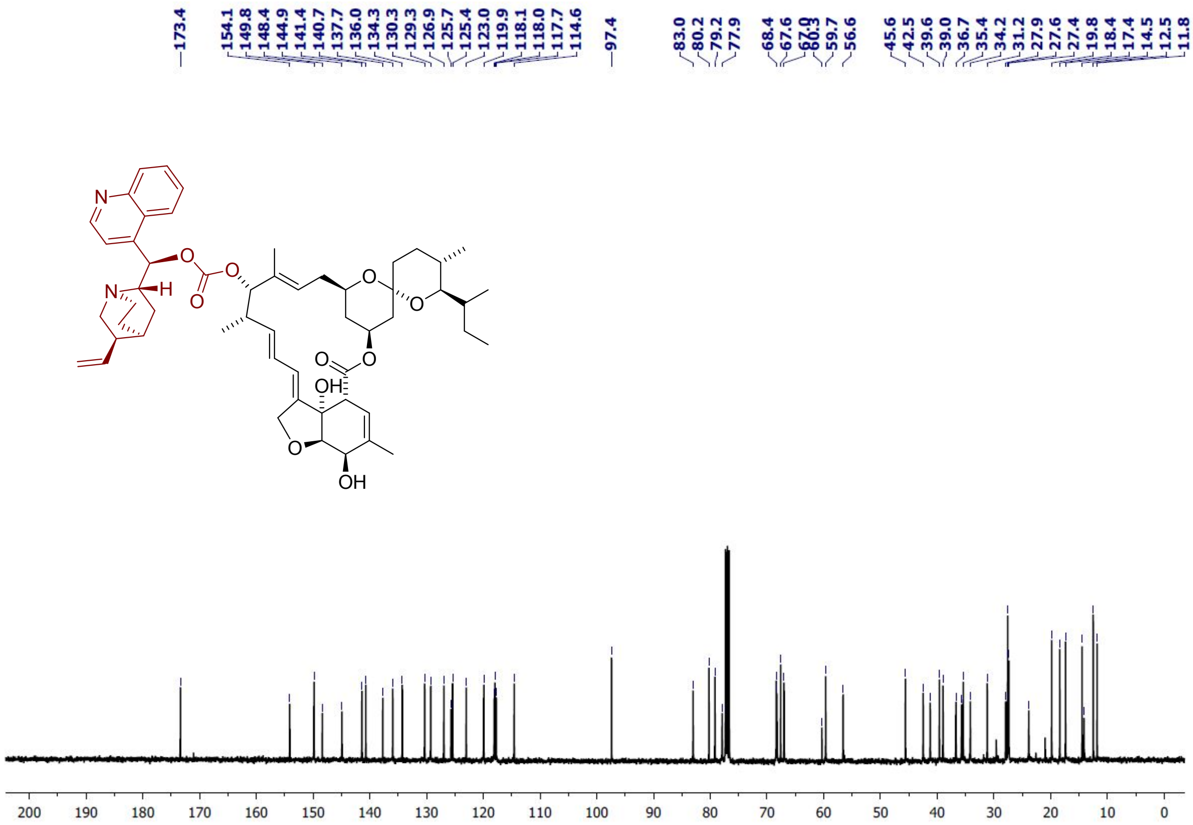

Figure S7. The $^{13}C\{^{1}H\}$ NMR (101 MHz) spectrum of **7** in chloroform-d.

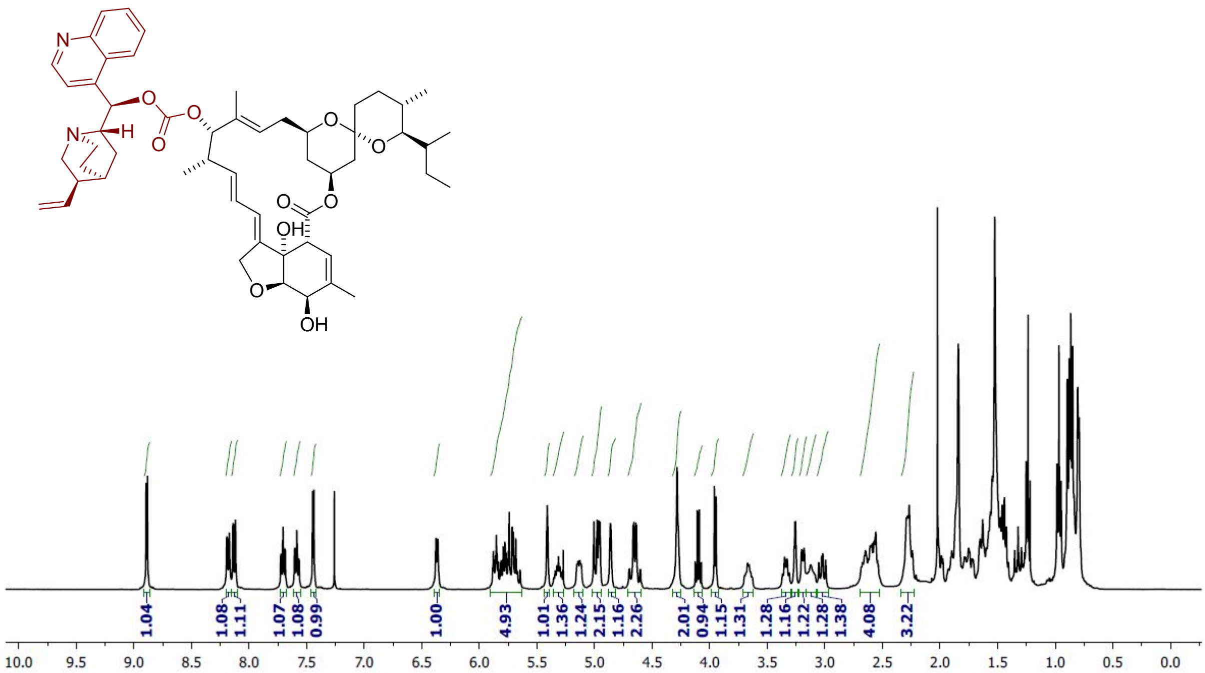

Figure S8. The ^{1}H NMR (400 MHz) spectrum of **7** in chloroform-d.

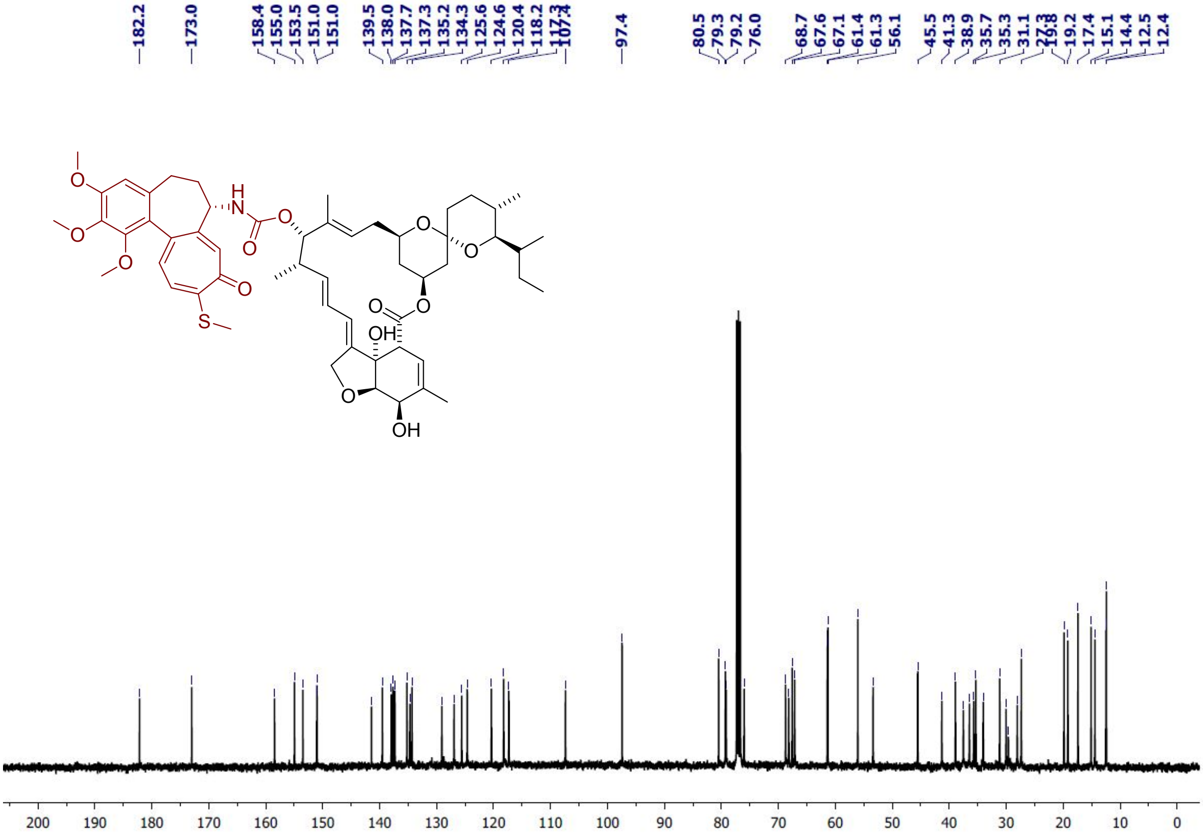

Figure S9. The ${}^{13}C\{{}^{1}H\}$ NMR (101 MHz) spectrum of **8** in chloroform-d.

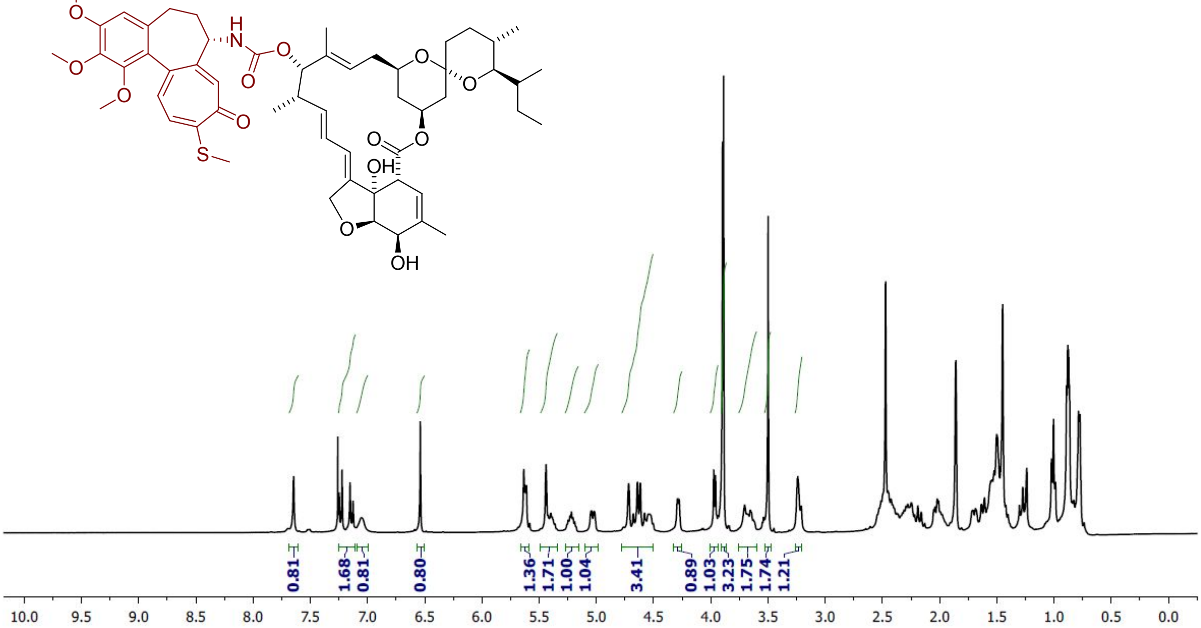

Figure S10. The ${}^{1}H$ NMR (400 MHz) spectrum of **8** in chloroform-d.

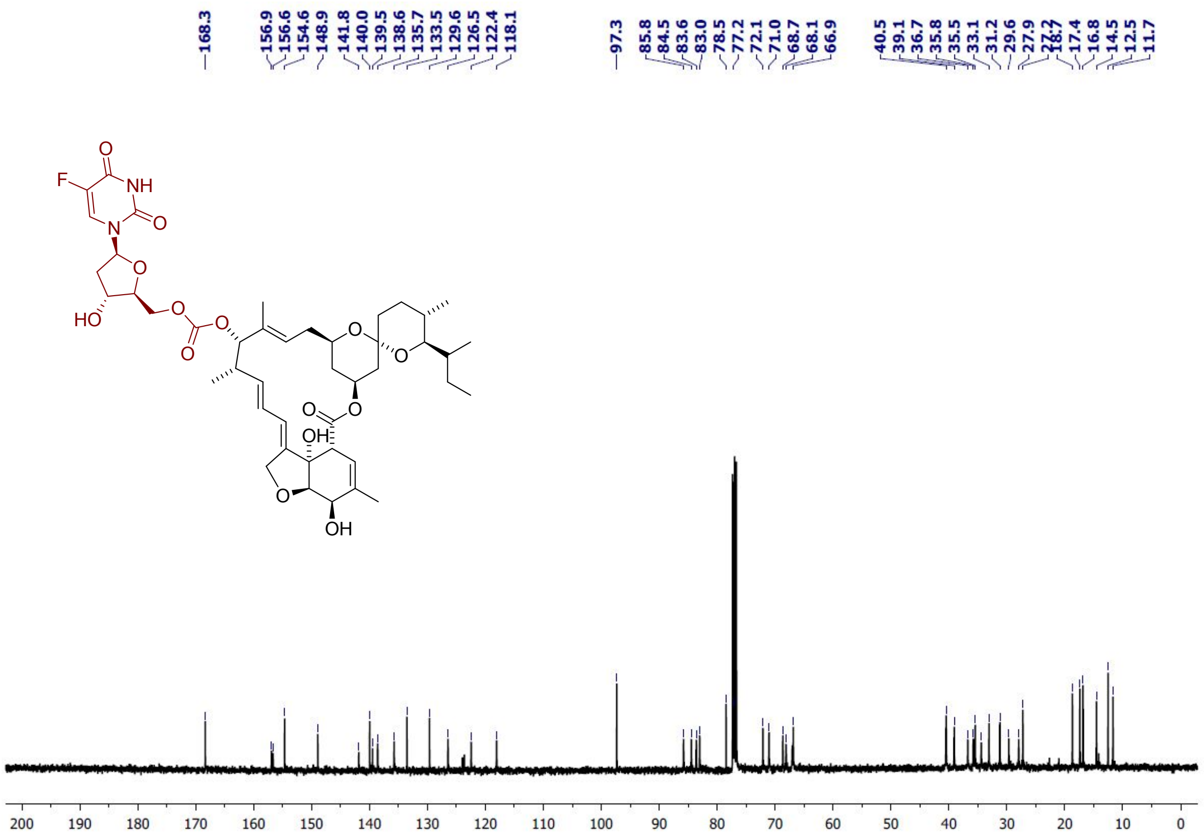

Figure S11. The $^{13}C\{^{1}H\}$ NMR (101 MHz) spectrum of **10** in chloroform-d.

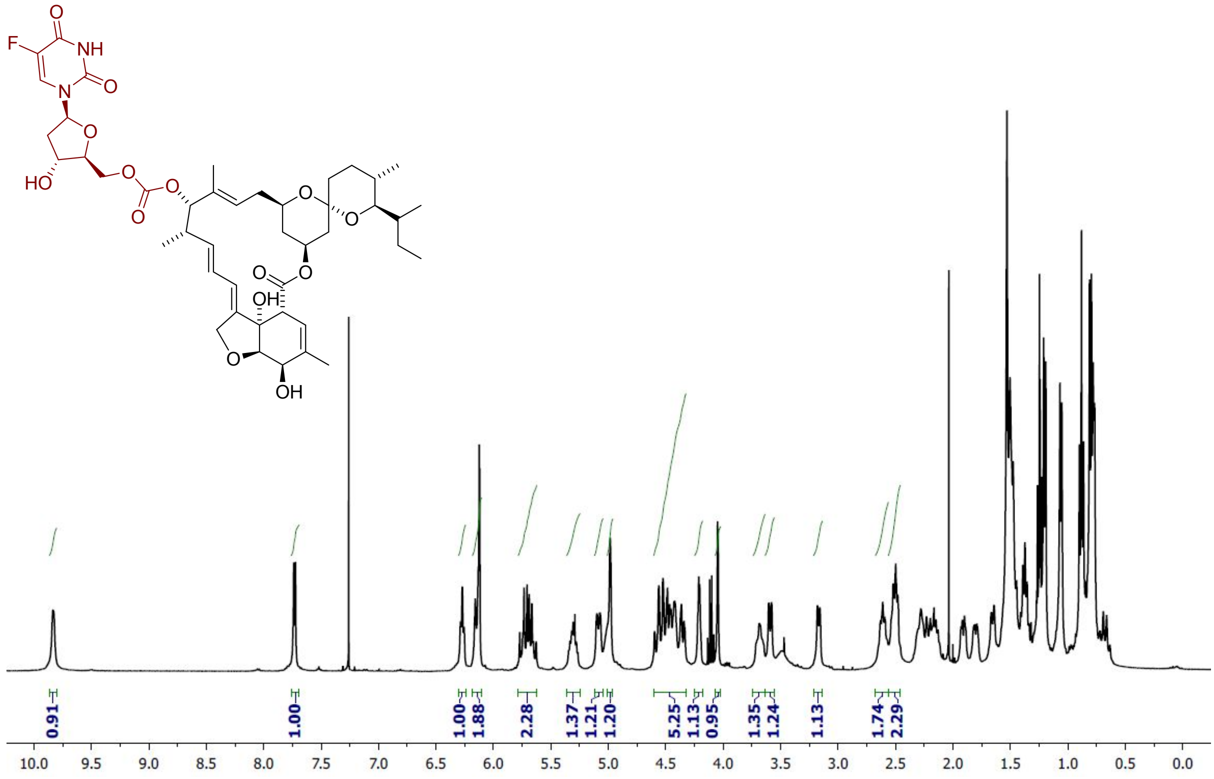

Figure S12. The ^{1}H NMR (400 MHz) spectrum of **10** in chloroform-d.

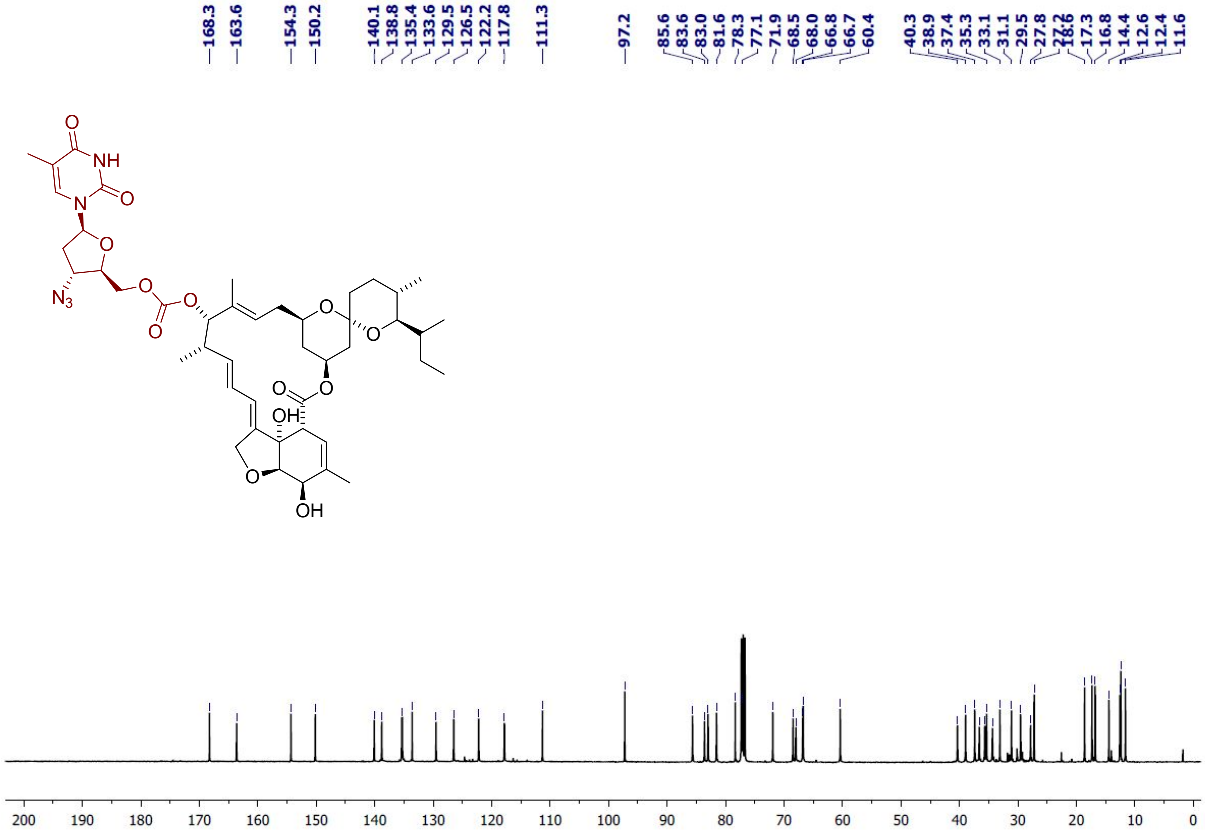

Figure S13. The $^{13}C\{^{1}H\}$ NMR (101 MHz) spectrum of **11** in chloroform-d.

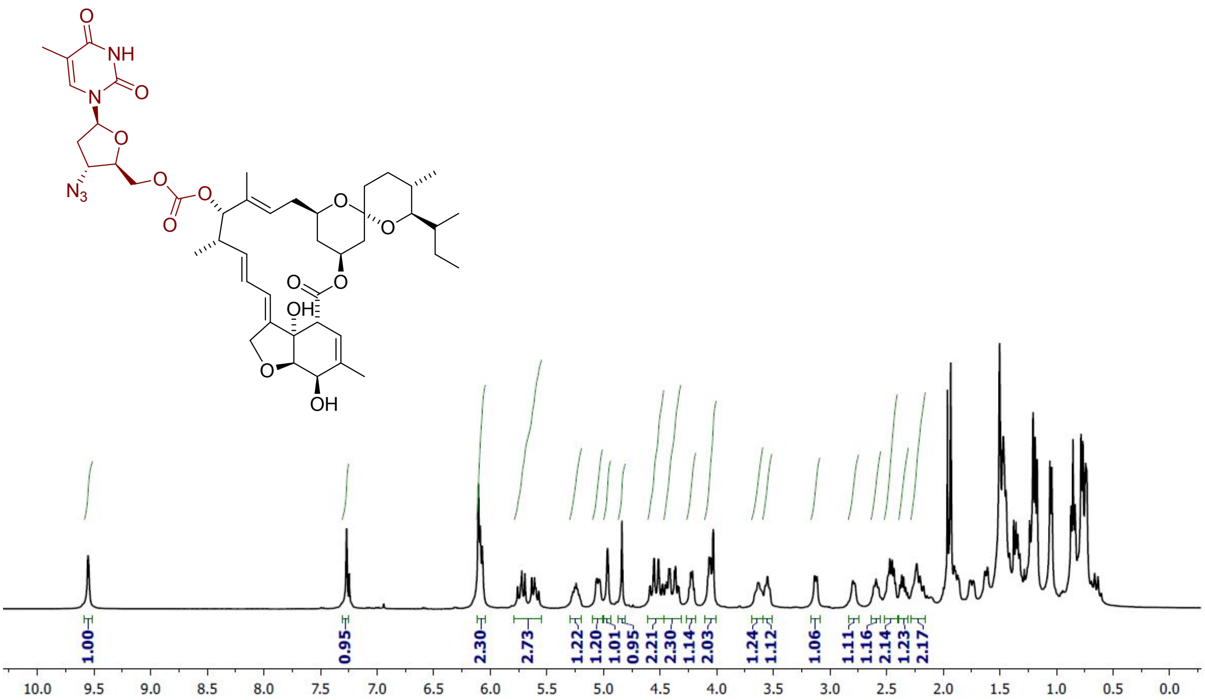

Figure S14. The ^{1}H NMR (400 MHz) spectrum of **11** in chloroform-d.

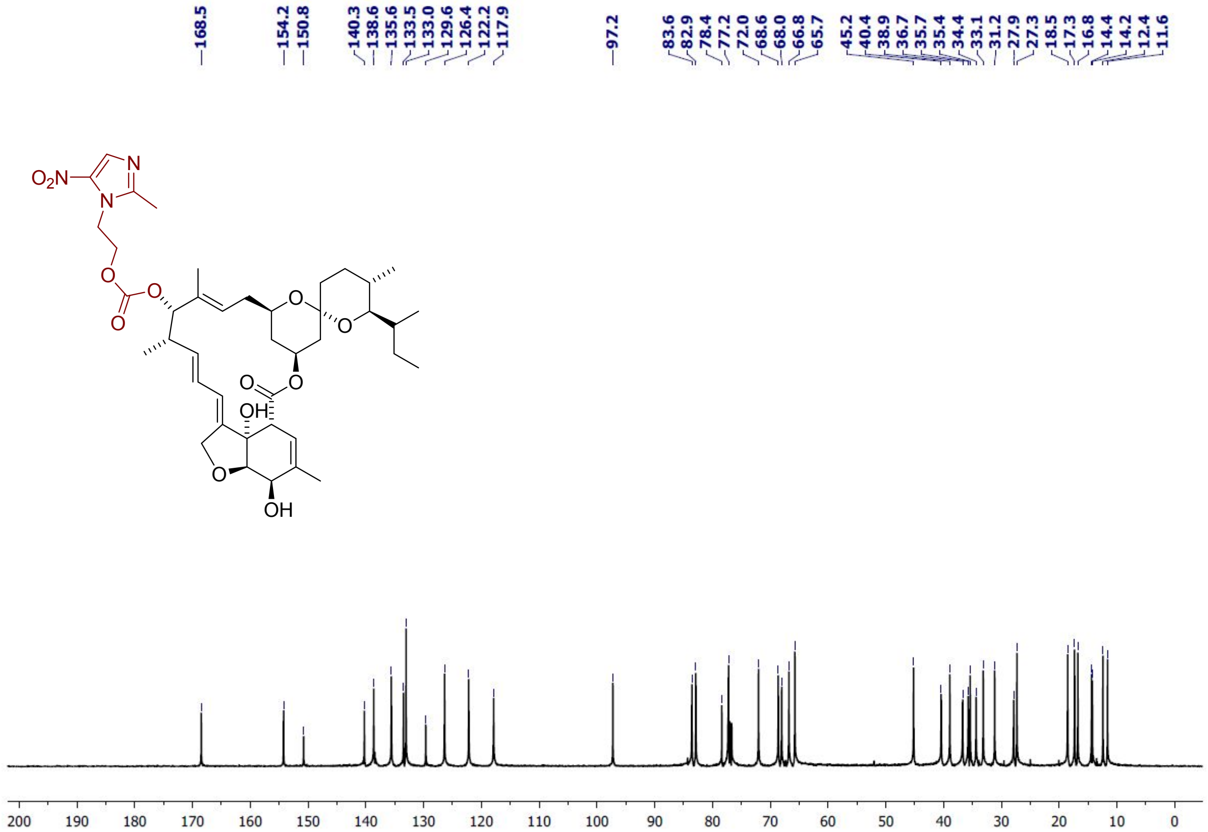

Figure S15. The $^{13}C\{^{1}H\}$ NMR (101 MHz) spectrum of **12** in chloroform-d.

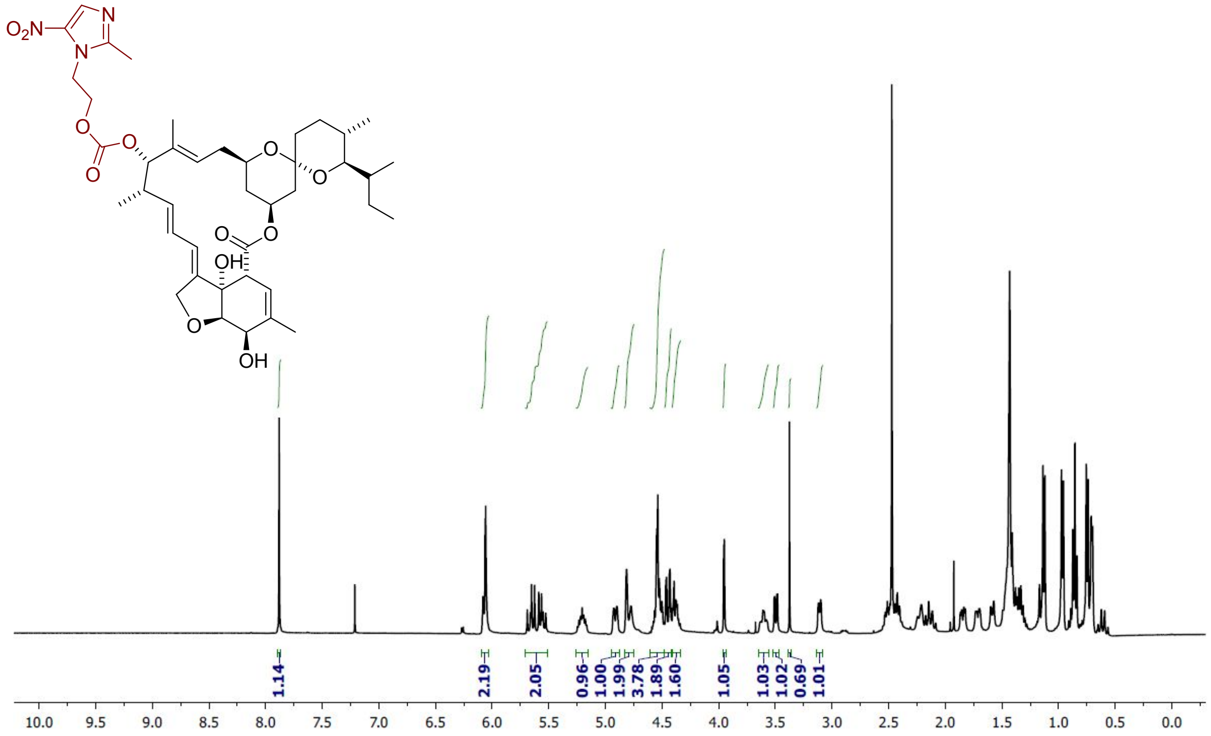

Figure S16. The ^{1}H NMR (400 MHz) spectrum of **12** in chloroform-d.

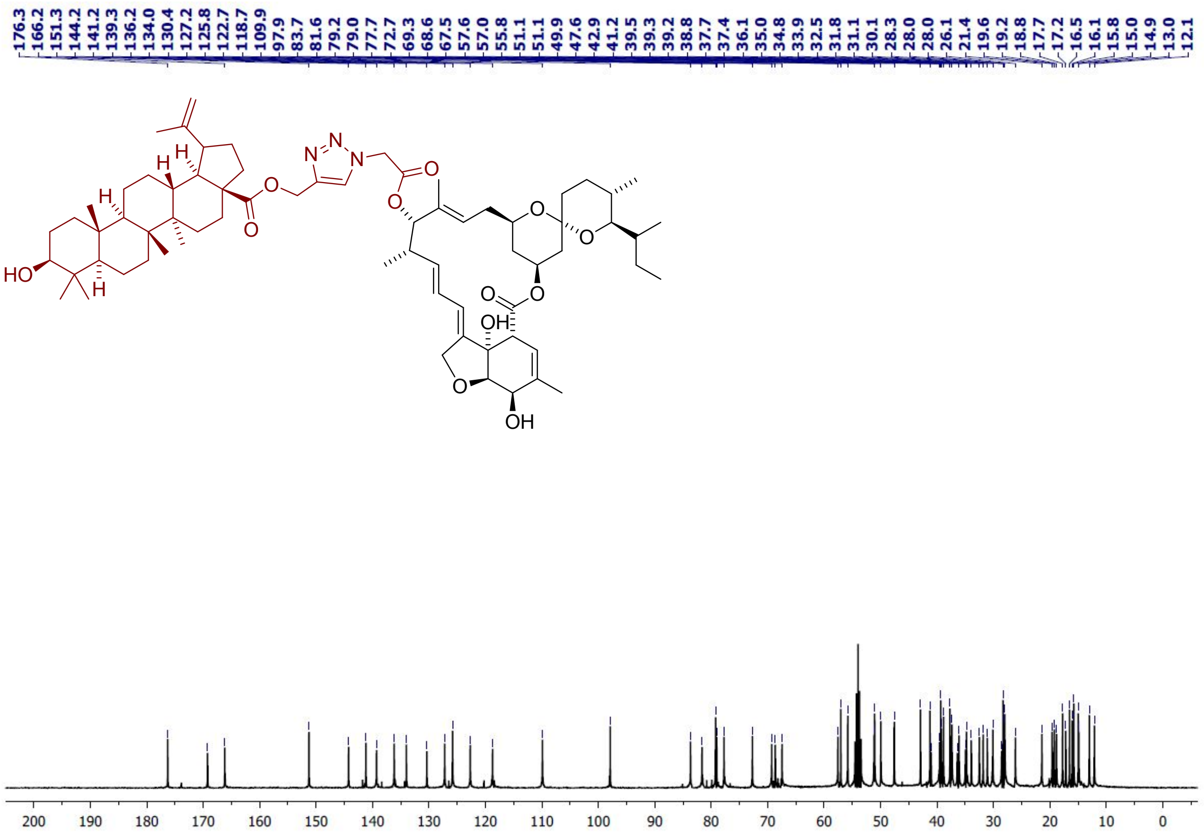

Figure S17. The $^{13}C\{^1H\}$ NMR (101 MHz) spectrum of **15** in methylene chloride-d2.

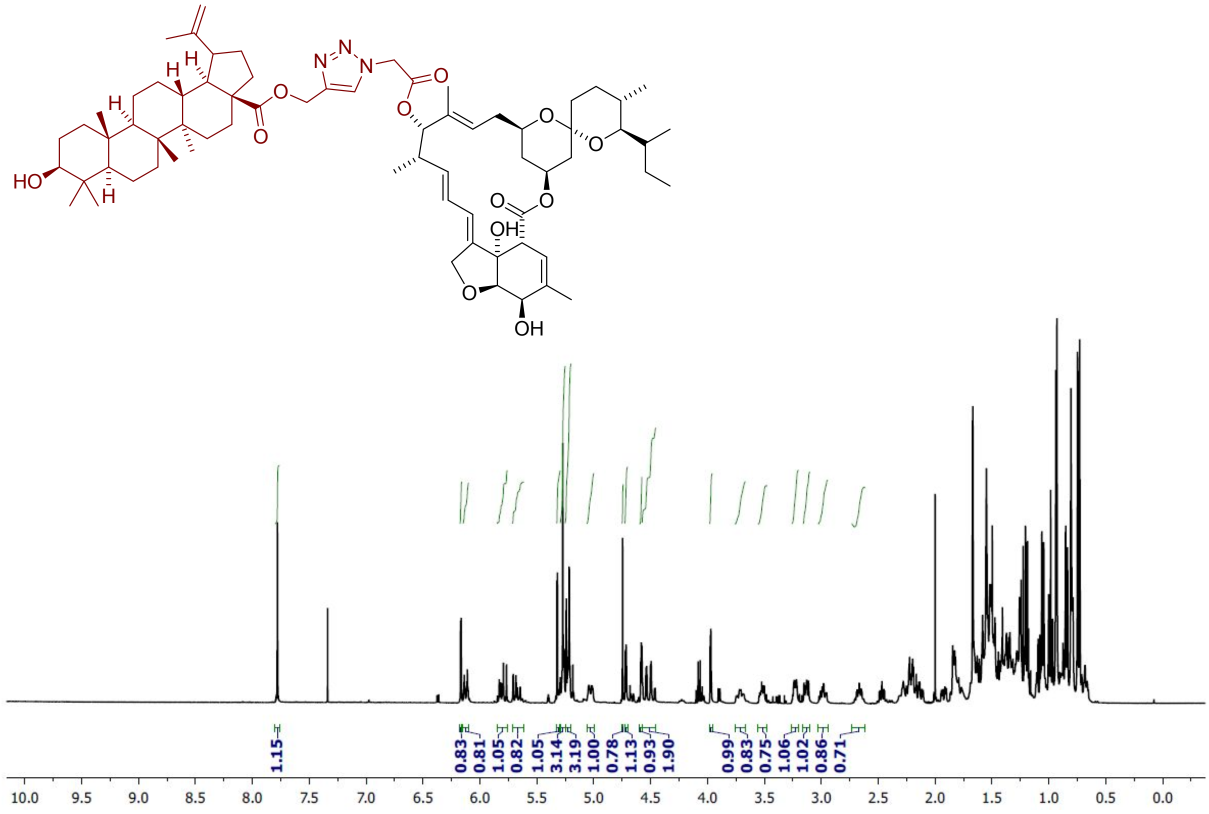

Figure S18. The ^1H NMR (400 MHz) spectrum of **15** in methylene chloride-d2.

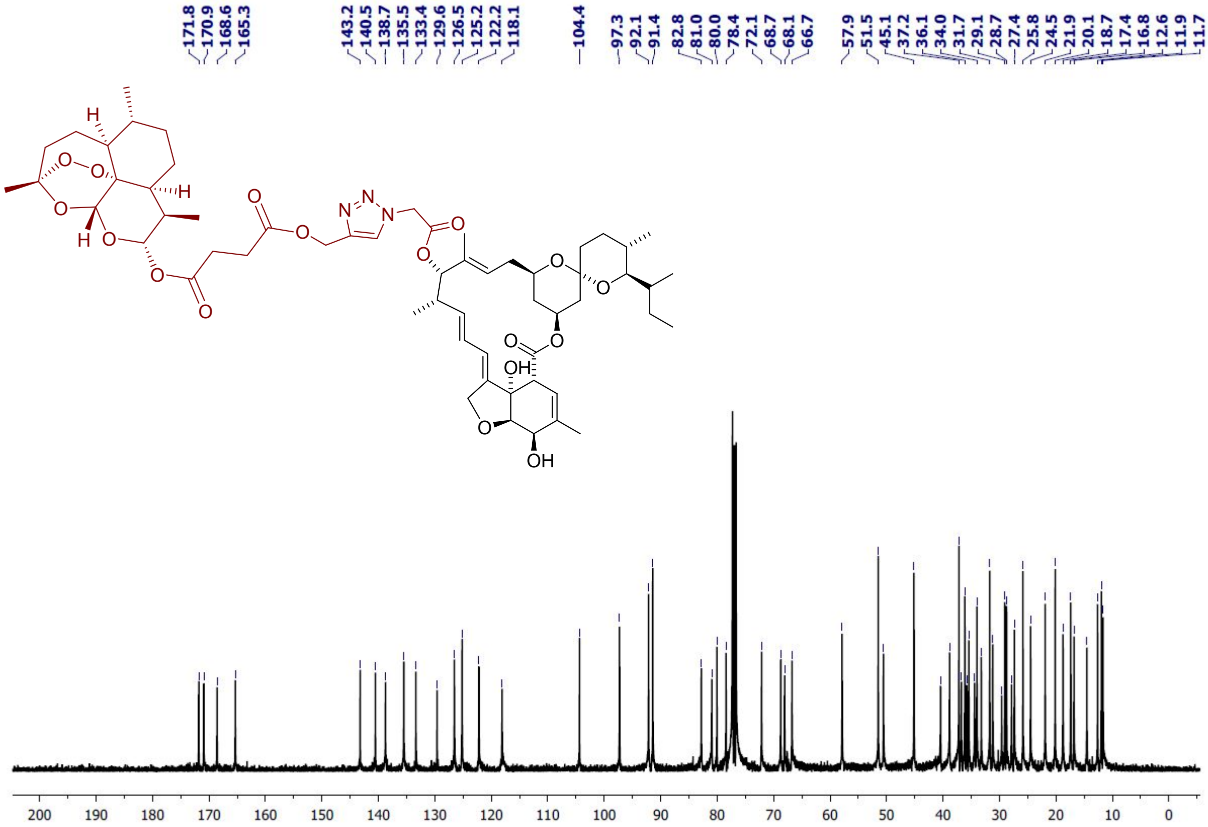

Figure S19. The $^{13}C\{^1H\}$ NMR (101 MHz) spectrum of **16** in chloroform-d.

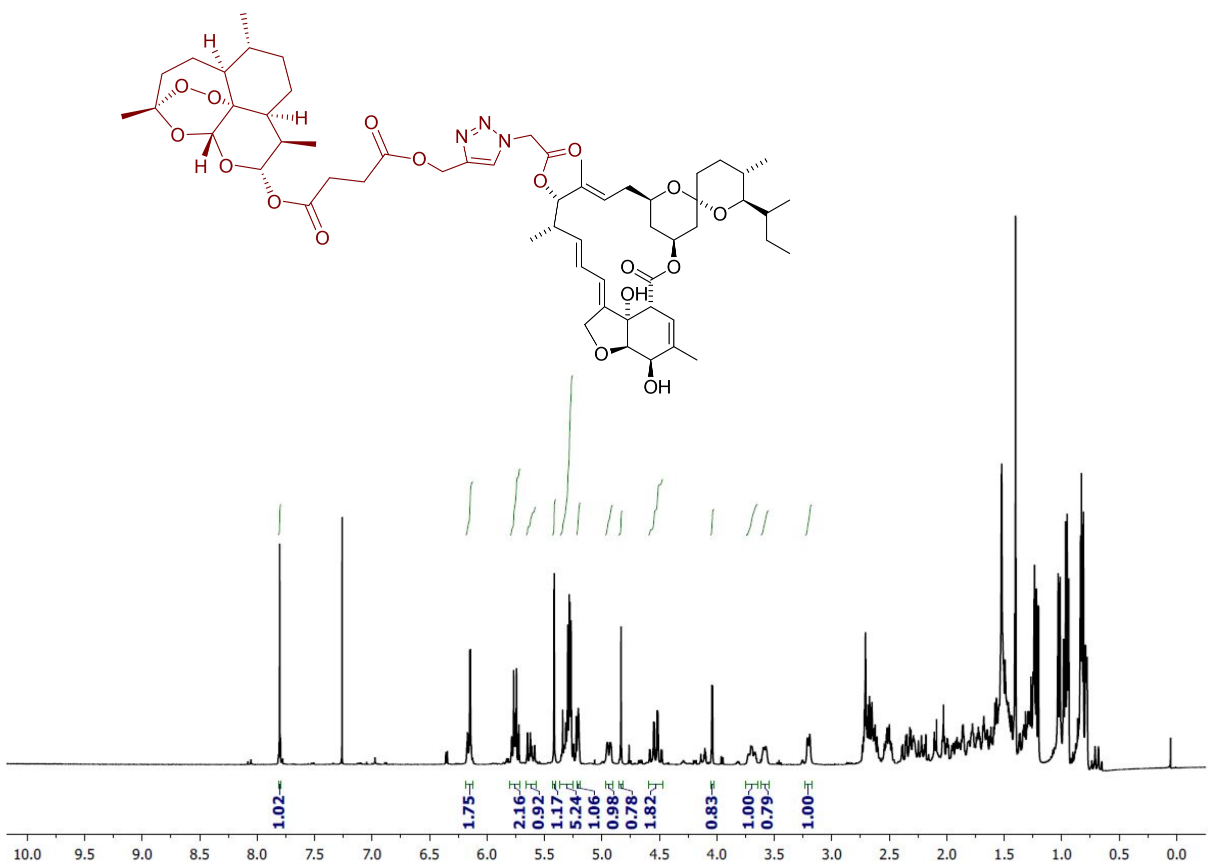

Figure S20. The ^1H NMR (400 MHz) spectrum of **16** in chloroform-d.

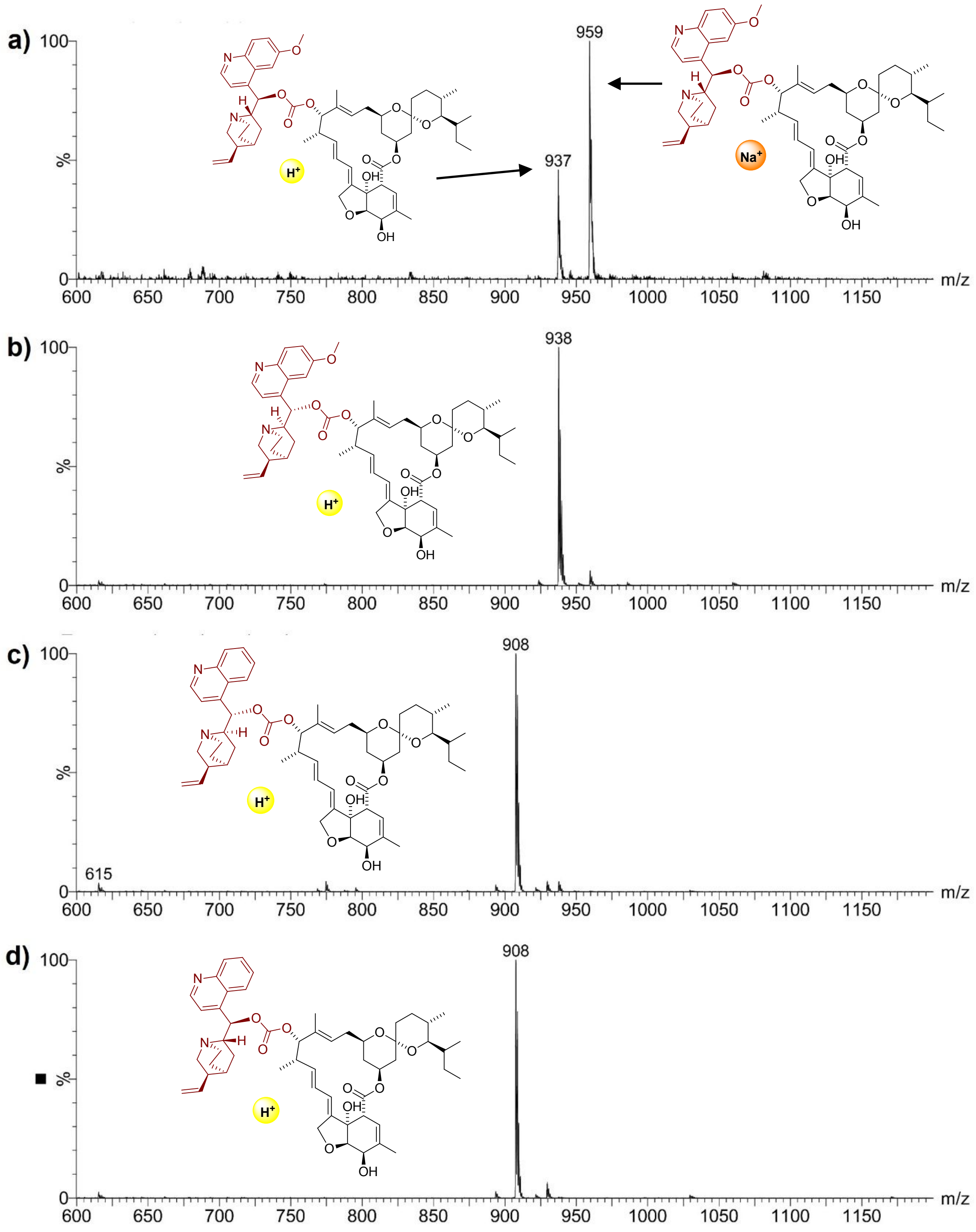

Figure S21. The ESI mass spectra of a mixture of **4** (a), **5** (b), **6** (c), and **7** (d), with $NaClO_4$ at cv = 10 V.

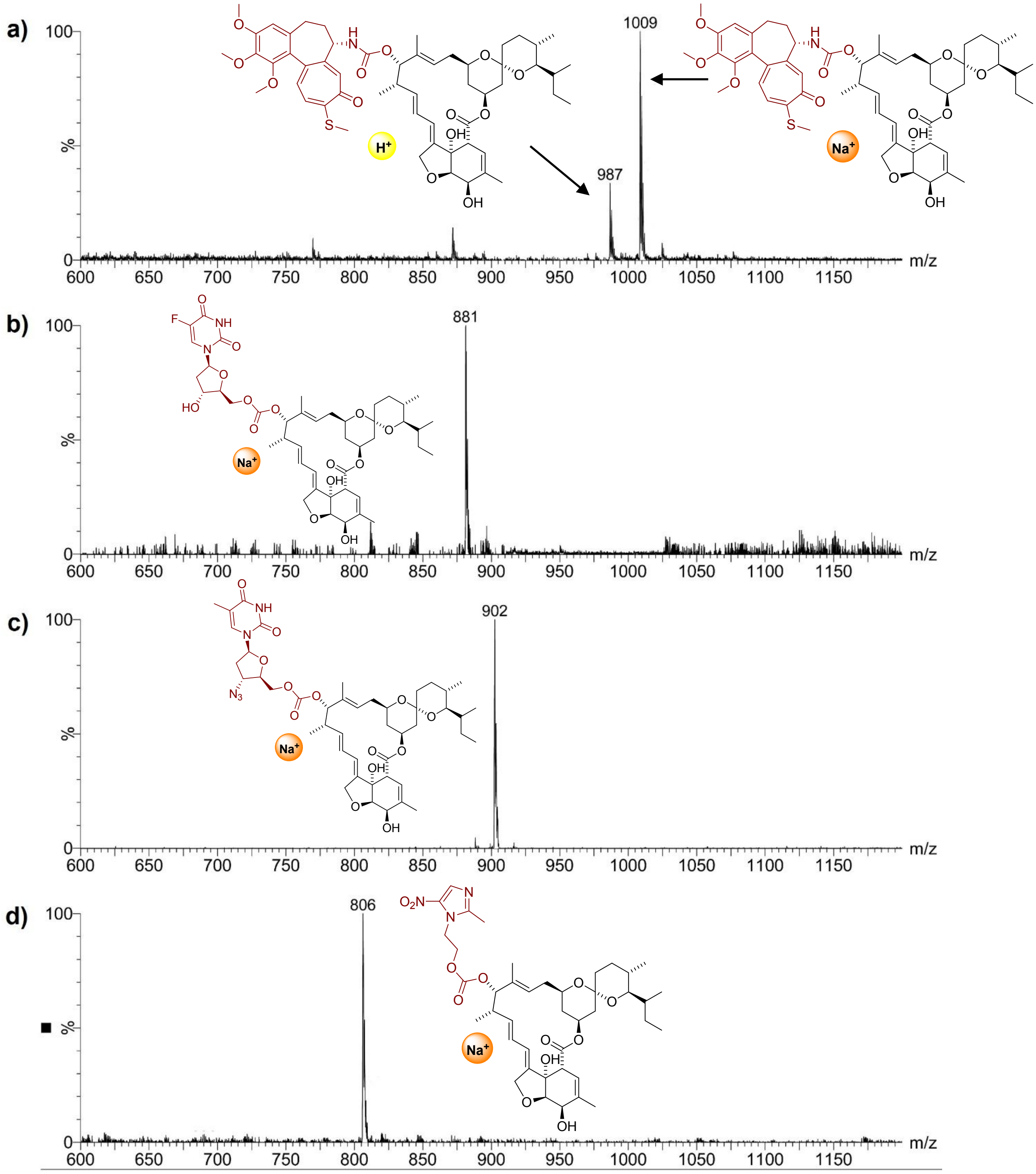

Figure S22. The ESI mass spectra of a mixture of **8** (a), **10** (b), **11** (c), and **12** (d), with $NaClO_4$ at cv = 10 V.

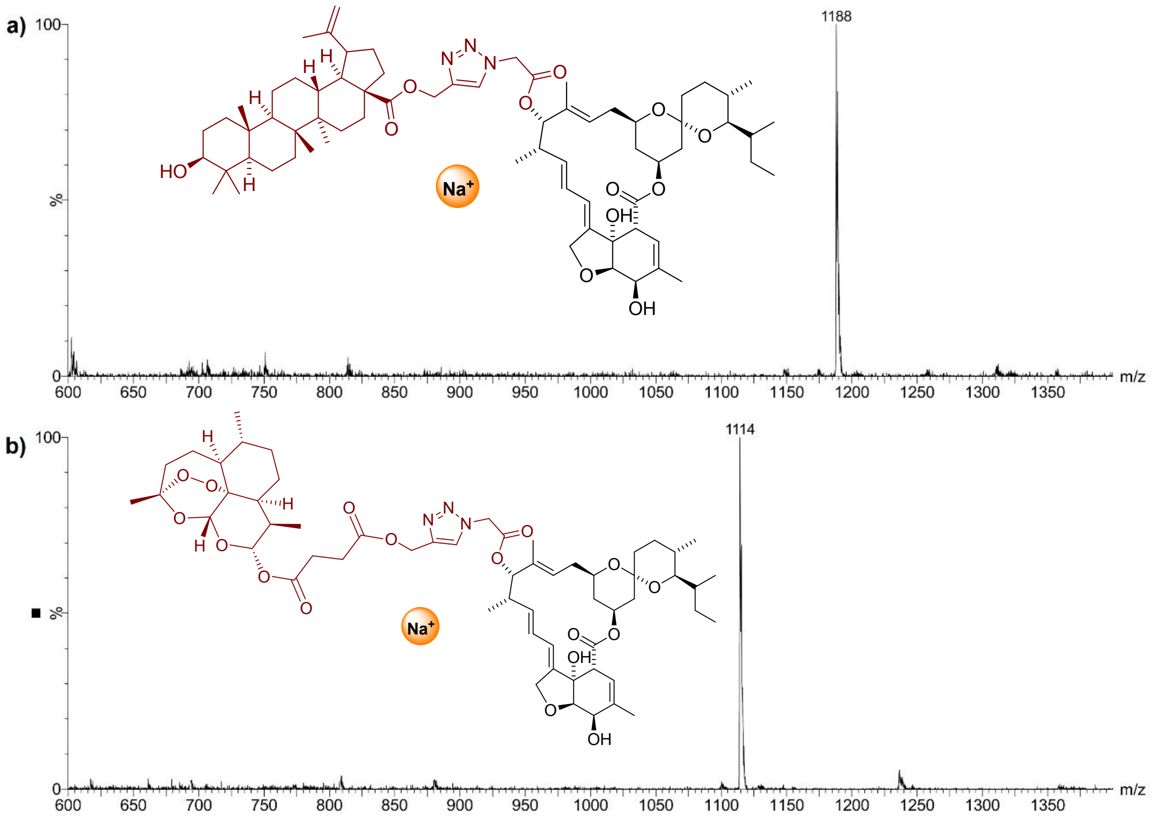

Figure S23. The ESI mass spectra of a mixture of **15** (a), and **16** (b) with $NaClO_4$ at cv = 10 V.
